# Supplementary material for: Single-cell transcriptional changes associated with drug tolerance and response to combination therapies in cancer
Source: Nat Commun. 2021 Mar 12;12:1628. doi: 10.1038/s41467-021-21884-z (PMC7955121; doi:10.1038/s41467-021-21884-z)
Supplement: Supplementary file 1 — Supplementary Information [file 41467_2021_21884_MOESM1_ESM.pdf]

Single-cell transcriptional changes associated with drug tolerance and response to combination therapies in cancer

Alexandre F. Aissa[1], Abul B.M.M.K. Islam[1, 2], Majd M. Ariss[1], Cammille C. Go[1], Alexandra E. Rader[1], Ryan D. Conrardy[1], Alexa M. Gajda[1], Carlota Rubio-Perez[3], Klara Valyi-Nagy[4], Mary Pasquinelli[5], Lawrence E. Feldman[5], Stefan J. Green[6], Nuria Lopez-Bigas[3], Maxim V. Frolov[1] and Elizaveta V. Benevolenskaya[1*].

[1] Department of Biochemistry and Molecular Genetics, University of Illinois at Chicago 900 S. Ashland Ave, Chicago 60607, USA
[2] Department of Genetic Engineering and Biotechnology, University of Dhaka, Dhaka 1000, Bangladesh
[3] Biomedical Genomics Lab, Institute for Research in Biomedicine (IRB), Barcelona 08003, Spain
[4] Department of Pathology, University of Illinois at Chicago, 840 South Wood Street, Chicago, IL, 60612, USA
[5] Department of Medicine, Section of Hematology/Oncology, University of Illinois at Chicago, 840 South Wood Street, Chicago, IL, 60612, USA
[6] Genome Research Core, Research Resources Center, University of Illinois at Chicago

[*] Corresponding author, e-mail: evb@uic.edu

Supplementary Figures

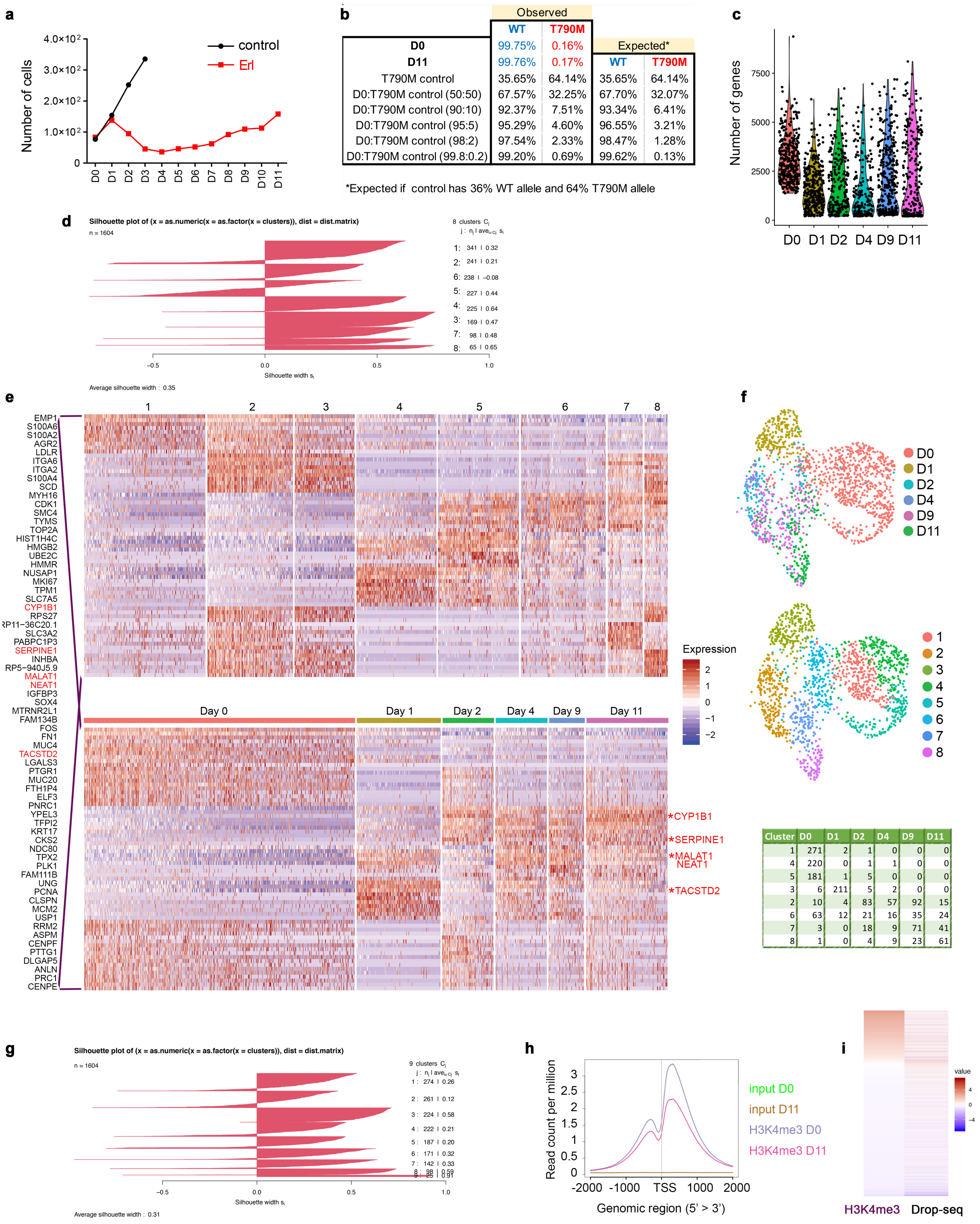

b

	Observed WT	Observed T790M	Expected* WT	Expected* T790M
D0	99.75%	0.16%		
D11	99.76%	0.17%		
T790M control	35.65%	64.14%	35.65%	64.14%
D0:T790M control (50:50)	67.57%	32.25%	67.70%	32.07%
D0:T790M control (90:10)	92.37%	7.51%	93.34%	6.41%
D0:T790M control (95:5)	95.29%	4.60%	96.55%	3.21%
D0:T790M control (98:2)	97.54%	2.33%	98.47%	1.28%
D0:T790M control (99.8:0.2)	99.20%	0.69%	99.62%	0.13%

*Expected if control has 36% WT allele and 64% T790M allele

f

Cluster	D0	D1	D2	D4	D9	D11
1	271	2	1	0	0	0
4	220	0	1	1	0	0
5	181	1	5	0	0	0
3	6	211	5	2	0	0
2	10	4	83	57	92	15
6	63	12	21	16	35	24
7	3	0	18	9	71	41
8	1	0	4	9	23	61

Supplementary Fig. 1. Assignments to tolerant states. a, Growth curve of PC9 cells treated with erlotinib (Erl, 2 μM) during 11 days (D0-D11), and growth curve of PC9 cells without addition of erlotinib during 3 days (D1, D2, and D3). This consecutive measurement experiment was performed in parallel to the plate for cell counting using Hoechst (Fig. 1a). Drug-containing media was renewed at day 3, day 6 and day 9. At indicated timepoints, cells were counted using brightfield microscopy at the same well position during the whole period of drug treatment consecutively. Cell counts from three fields per well were combined; mean values are shown for *n* = 2 replicate wells. **b**, The frequency of T790M allele in PC9 cells as determined by PCR-assisted sequencing. Allelic frequencies in untreated PC9 cells (D0) and cells treated with 2 μM erlotinib for 11 days (D11) are compared to that of the EGFR-T790M mutant H1975 cells (T790M control). The different dilutions of PC9 with the H1975 control show the level of detection goes to <0.2%, which is the frequency of detecting T790M allele in PC9 cells. **c**, Distribution of the number of sequenced genes per cell, per sample, in untreated and erlotinib-treated conditions. Shown are the cells with at least 350 detected genes. **d**, Silhouette widths calculated for each cluster in Fig. 1e and the resulting average silhouette width. The applied resolution was 0.55. **e**, Heatmap of top cluster markers across clusters and days of treatment. Selected markers, *CYP1B1, SERPINE1, NEAT1, MALAT1* and *TACSTD2*, are labeled. **f**, Regressing cell cycle genes does not change attribution of DT states. Top markers for S-phase were *PCNA, MCMs, CDT1, CCNE2, ORC6, CDC6*, for S G2/M-phase were *RPA3* and *LIG4*, and for G2/M-phase were *CDK1*, *CDC25B*, *CDC25C*, *CCNA2*, *CCNB1*, *CCNB2* and *PLK*. UMAP representation of data for PC9 cells as Fig. 1e but after regressing cell cycle genes; colors are according to days of treatment (*top panel*) or clusters (*middle panel*). The number of cells from each sample is listed for each cluster in the Table *below*. **g**, Silhouette widths calculated for each cluster in Supplementary Fig. 1f and the resulting average silhouette width. Cluster numeration is different from that in (f). **h**, Average enrichment of H3K4me3 across TSS regions. Because the number of detected genes per cell was higher in untreated condition (c), to test whether this is a technical artifact or different transcriptional robustness, we analyzed H3K4me3, the histone modification associated with active transcription. ChIP-seq analysis using anti-H3K4me3 antibodies is presented for D0 and D11 cells compared with corresponding input samples. ChIP-seq showed a global decrease in H3K4me3 at the transcription start site (TSS) regions in treated cells. **i**, Genes increased in expression level commonly display an increase in H3K4me3 enrichment.

Genes differentially expressed in D11 versus D0 cells (Log2FoldChange>0.1) as determined by scRNA-seq were sorted by their enrichment in H3K4me3.

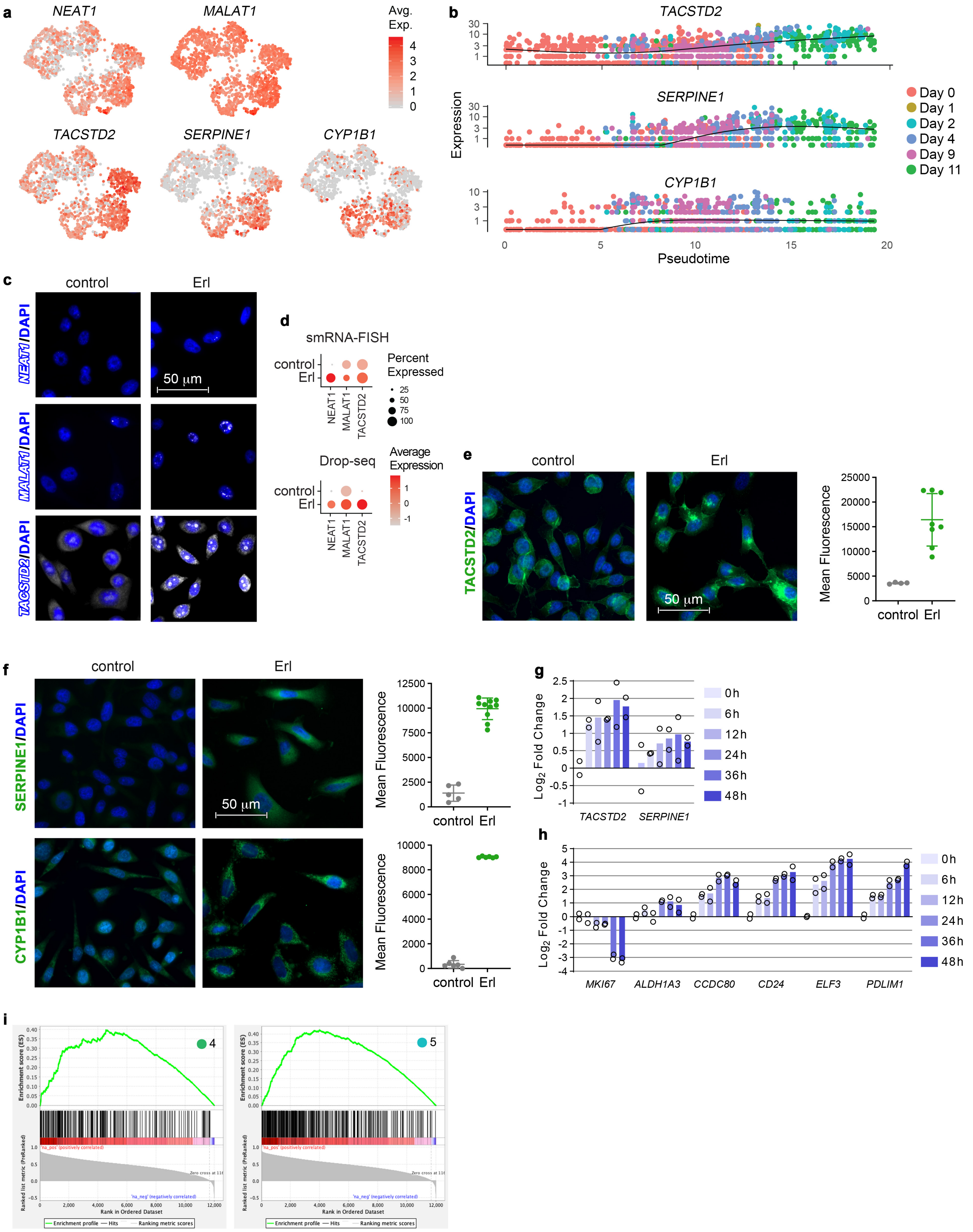
a
NEAT1
MALAT1
Avg. Exp.
4
3
2
1
0
TACSTD2
SERPINE1
CYP1B1
b
TACSTD2
SERPINE1
CYP1B1
Expression
Pseudotime
0
5
10
15
20
Day 0
Day 1
Day 2
Day 4
Day 9
Day 11
c
control
Erl
NEAT1/DAPI
MALAT1/DAPI
TACSTD2/DAPI
50 µm
d
smRNA-FISH
control
Erl
NEAT1
MALAT1
TACSTD2
Percent Expressed
25
50
75
100
Drop-seq
Average Expression
1
0
-1
e
control
Erl
TACSTD2/DAPI
50 µm
Mean Fluorescence
25000
20000
15000
10000
5000
0
f
control
Erl
SERPINE1/DAPI
CYP1B1/DAPI
50 µm
Mean Fluorescence
12500
10000
7500
5000
2500
0
8000
6000
4000
2000
g
Log2 Fold Change
TACSTD2
SERPINE1
0 h
6 h
12 h
24 h
36 h
48 h
h
MKI67
ALDH1A3
CCDC80
CD24
ELF3
PDLIM1
i
4
5
Enrichment score (ES)
Ranked list metric (PreRanked)
Rank in Ordered Dataset
'na_pos' (positively correlated)
'na_neg' (negatively correlated)
Enrichment profile
Hits
Ranking metric scores

Supplementary Fig. 2. Distribution of top markers of DT states. a, Feature plots projected onto UMAP showing the increased expression of selected top genes across the Seurat clusters. The UMAP is from Fig. 1e. **b**, Monocle analysis shows that expression of the three selected genes increases during erlotinib treatment. **c**, smRNA-FISH for top markers of tolerant states identified in Drop-seq, lncRNAs *NEAT1* and *MALAT1*, and protein-encoding transcripts for *TACSTD2*, co-stained with DAPI (blue). PC9 cells were treated with erlotinib for 1 day and hybridized with *TACSTD2* probes, and for 2 days and hybridized with *NEAT1* and *MALAT1* probes. images were acquired using the Zeiss microscope, and are representatives of 2 independent experiments. **d**, Dot plot of transcript expression from smRNA-FISH and Drop-seq data. The number of transcripts from the smRNA-FISH was counted from n = 5 images (>100 cells), and is representative of two experiments. Percentage of cells expressing a transcript is represented by the size of the dot, where large dots correspond to a higher percentage of cells. Scaled average expression across cells is shown by the color of the dot, with brighter red representing a high expression, and light grey representing low expression. **e**, TACSTD2 protein level is increased in cells treated with erlotinib (for 1 day) as evidenced by immunofluorescence with anti-TACSTD2 antibody. The immunostaining is in green, with DAPI (nuclear) counter stain (blue). **f**, Immunostaining for top markers SERPINE1 and CYP1B1 shows increased protein expression level in erlotinib-treated cells. PC9 cells were treated with erlotinib for 4 days or 11 days and immunostaining was performed with antibodies to SERPINE1 and CYP1B1, respectively. Quantitation of the immunofluorescence experiments in (e) and (f) is presented on the *right,* mean ± standard deviation (SD) for n = 4 images in control and n = 8 images in Erl for TACSTD2, n = 5 images in control and n = 10 images in Erl for SERPINE1, n = 6 images in both conditions for CYP1B1, and is a representative of three independent experiments. **g**, Gene expression changes in the levels of top markers *TACSTD2* and *SERPINE1* using bulk RNA samples at early time points of erlotinib treatment. **h**, Gene expression changes in the levels of markers of early tolerant state. The RT-qPCR values in (g) and (h) were normalized to *POLR2B* level and presented as Log_2 fold changes relative to DMSO-treated control cells, with mean values for n = 2 biological replicates. **i**, The results of the GSEA run on the PC9 markers with the pre-ranked list of Spearman Correlation CCLE data. Cluster 4 (N=244): Normalized Enrichment Score (NES)=1.89, FDR q-value=0; Cluster 5 (N=358): NES=2.05, FDR q-value=0.

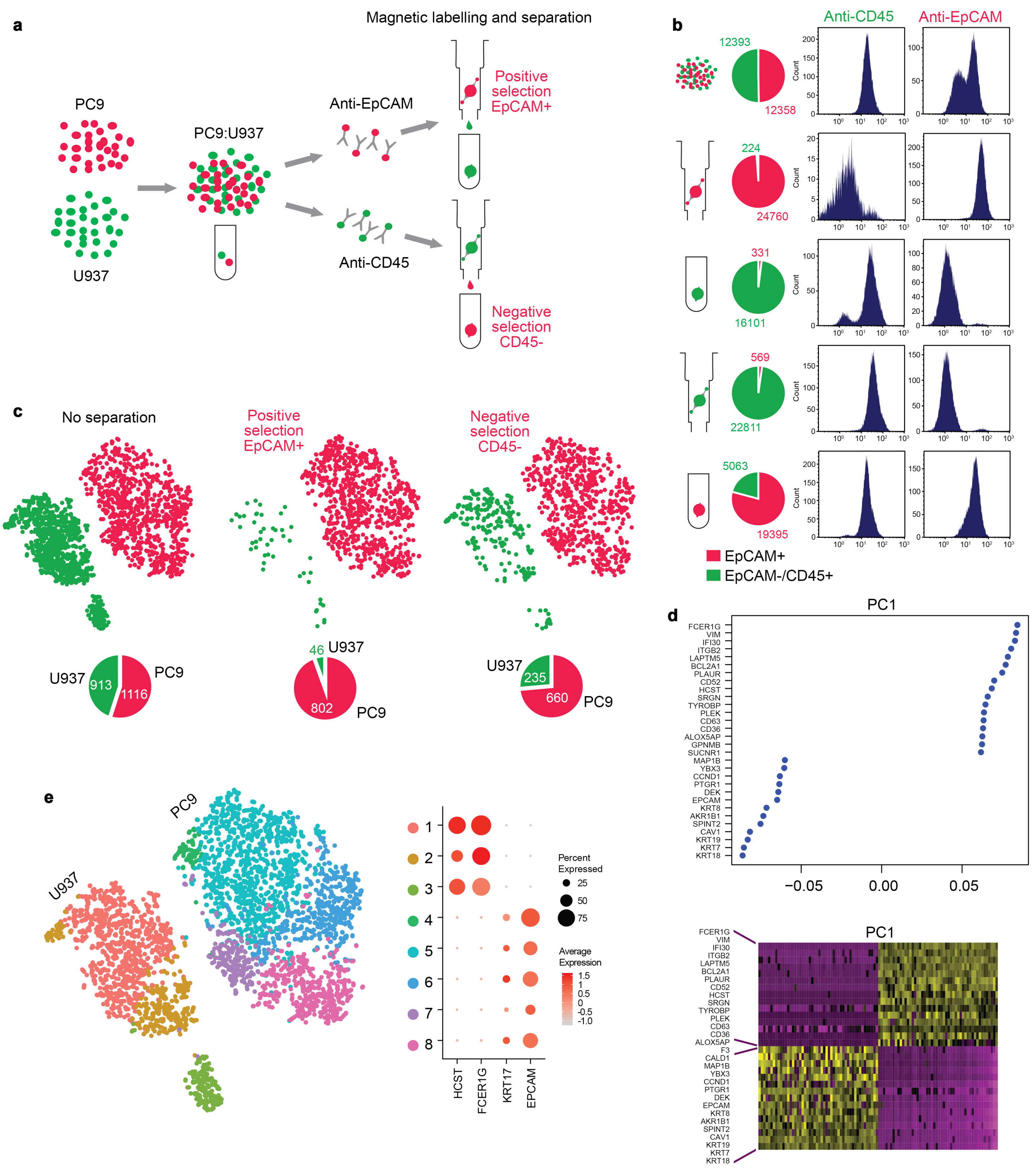

Supplementary Fig. 3. Drop-seq separates different cancer cell lines and identifies cell subpopulations.

a, Cell separation setup. Three samples were generated as following: The PC9:U937 sample comprised of PC9 cells mixed with differentiated U937 cells. Two similar samples were subjected to additional magnetic cell

sorting using antibodies for the cell surface markers EpCAM or CD45. Sorting resulted in the creation of EpCAM-positive ($EpCAM^+$) and CD45-negative ($CD45^-$) cell samples. **b**, Analysis of the mixtures of PC9 and U937 cells by flow cytometry reveals cells of the unrelated cell line after both positive and negative selection. Pie charts show numbers of $EpCAM^+$ (red) and EpCAM-negative/CD45-positve ($EpCAM^-$/$CD45^+$ green) in each sample, representing PC9 and U937 cells, respectively. **c**, t-SNE representation in Seurat distinguishes two distinct cell lines (green for U937 and red for PC9) within a single sample. Cell distribution of EpCAM-positive and CD45-negative samples reveals remaining cells of another cell line. Pie charts show cell number for each cell type counted in Seurat. **d**, Principal component (PC) 1 genes obtained with Seurat on the PC9:U937 sample showing the distribution of top 30 genes (*on the top*). Heatmap shows the relative expression level of the genes (rows) in 100 cells (columns) (*at the bottom*). **e**, A single t-SNE plot represents the eight cell clusters after merging the three samples (as in (c)) in Seurat. Cell clusters are distinguished by color. The plot separates clusters of U937 (Clusters 1-3) and PC9 cells (Clusters 4-8). The dot plot of average cluster gene expression (*on the right*) reflects top canonical markers that are differentially expressed between PC9 and U937 cells.

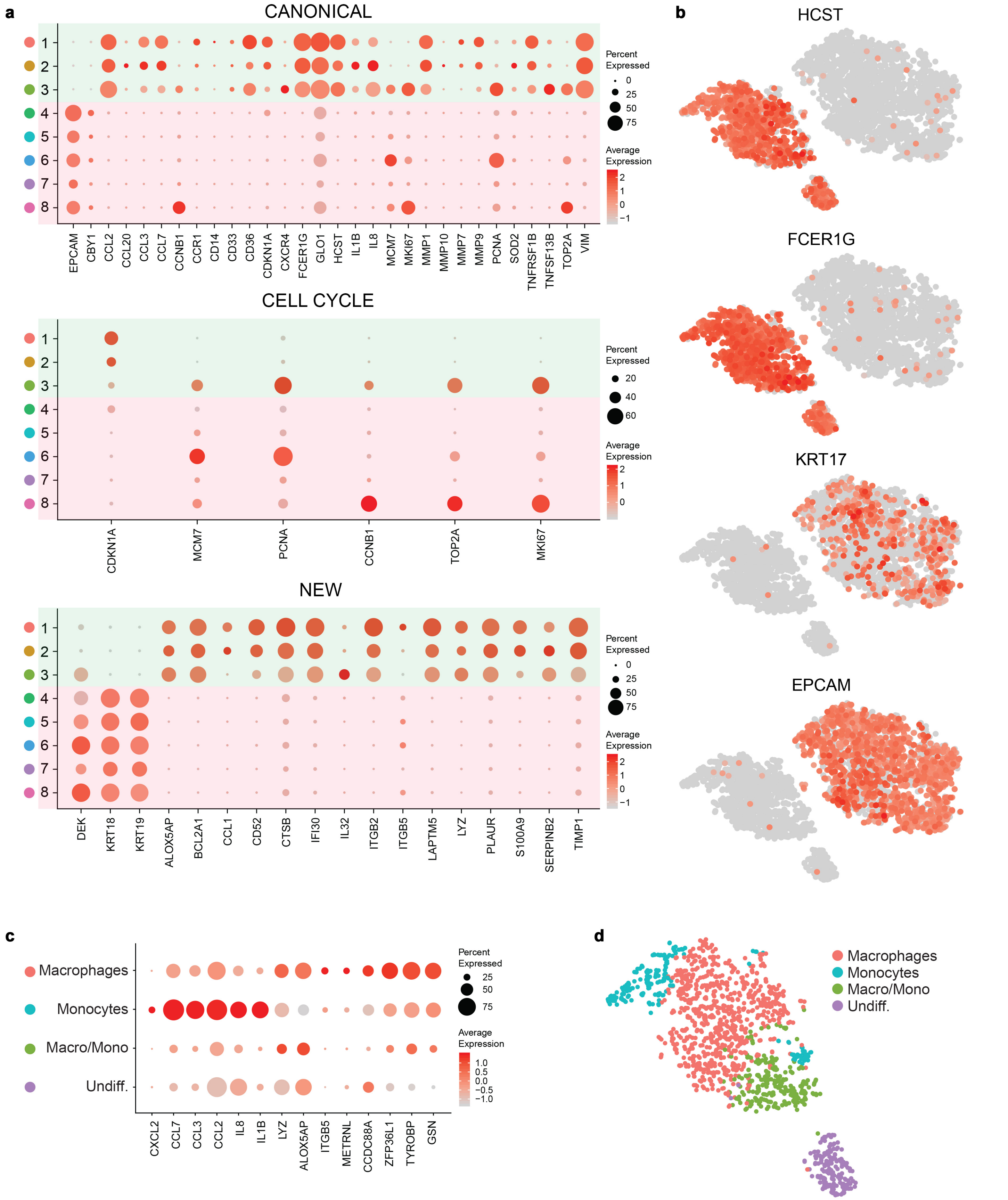
a
CANONICAL
1
2
3
4
5
6
7
8
EPCAM
CBY1
CCL2
CCL20
CCL3
CCL7
CCNB1
CCR1
CD14
CD33
CD36
CDKN1A
CXCR4
FCER1G
GLO1
HCST
IL1B
IL8
MCM7
MKI67
MMP1
MMP10
MMP7
MMP9
PCNA
SOD2
TNFRSF1B
TNFSF13B
TOP2A
VIM
Percent Expressed
0
25
50
75
Average Expression
2
1
0
−1
CELL CYCLE
CDKN1A
MCM7
PCNA
CCNB1
TOP2A
MKI67
Percent Expressed
20
40
60
Average Expression
2
1
0
NEW
DEK
KRT18
KRT19
ALOX5AP
BCL2A1
CCL1
CD52
CTSB
IFI30
IL32
ITGB2
ITGB5
LAPTM5
LYZ
PLAUR
S100A9
SERPINB2
TIMP1
Percent Expressed
0
25
50
75
Average Expression
2
1
0
−1
b
HCST
FCER1G
KRT17
EPCAM
c
Macrophages
Monocytes
Macro/Mono
Undiff.
CXCL2
CCL7
CCL3
CCL2
IL8
IL1B
LYZ
ALOX5AP
ITGB5
METRNL
CCDC88A
ZFP36L1
TYROBP
GSN
Percent Expressed
25
50
75
Average Expression
1.0
0.5
0.0
−0.5
−1.0
d
Macrophages
Monocytes
Macro/Mono
Undiff.

Supplementary Fig. 4. Top differentially expressed genes across the clusters of mixed PC9 and U937 cells. a, Dot plot of differentially expressed genes across the clusters (see also Supplementary Data 9). Canonical U937 markers are shown in comparison to the PC9 marker characteristic of epithelial cells *EpCAM*. Cell cycle genes are among top differentially expressed genes. Drop-seq also identifies highly expressed genes with differential expression in U937 cells compared to PC9 cells, which were not described previously. **b**, t-SNE depicting canonical hematopoietic (*FCER1G*, *HCST*) and epithelial (*KRT17, EPCAM*) markers across the clusters. **c** and **d** U937 cell clusters correspond to macrophages and monocytes. U937 cells were subset and re-clustered, separately from PC9 cells. Dot plot in (c) shows that expression of canonical markers of macrophages and monocytes (see Methods) differ in the identified four clusters. Each cluster was named accordingly: “Macrophages” express *TYROBP* and *GSN*; “Monocytes” express interleukins, chemokines, and *SOD2*; intermediate cells “Macro/Mono” express *LYZ* and *ALOX5AP* and mixed markers; and a population that failed to differentiate (“Undiff.”) lacks macrophage or monocyte markers and expresses high levels of cell cycle genes. (d) t-SNE on U937 cells depicts four clusters, each of which can be named according to expression of canonical macrophages and monocyte markers.

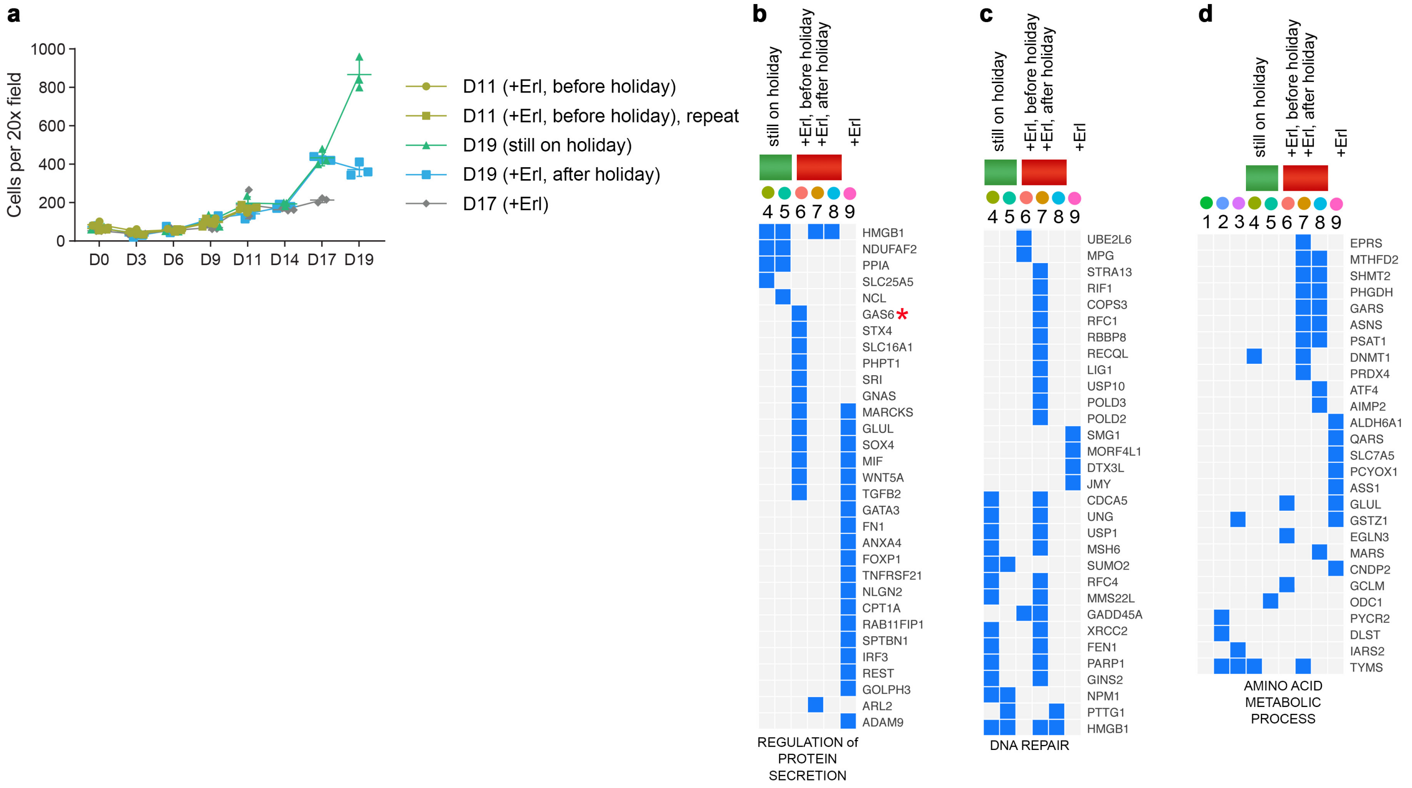

Supplementary Fig. 5. Drop-seq reveals biological processes and pathways associated with drug tolerance during drug holiday. a, Growth curve of PC9 cells subjected to drug holiday experiment. Cells were treated with Erl for 11 days, after which "D19" samples were withdrawn from erlotinib for 6 days until day 17th and then treated with DMSO and Erl, respectively, for another 2 days, before processing for Drop-seq. Cell counts are based on brightfield microscopy and presented as mean ± SD for n = 3 replicates. **b**, Top markers distinguishing treated and untreated cells are listed for GO term REGULATION_of_PROTEIN_SECRETION. The *GAS6* gene is indicated by asterisk. **c**, Top markers distinguishing treated and untreated cells are listed for GO term DNA_REPAIR. In (b) and (c), only genes preferentially expressed in treated cells compared to untreated are shown. **d**, Top markers distinguishing treated and untreated cells are listed for GO term AMINO_ACID_METABOLIC_PROCESS.

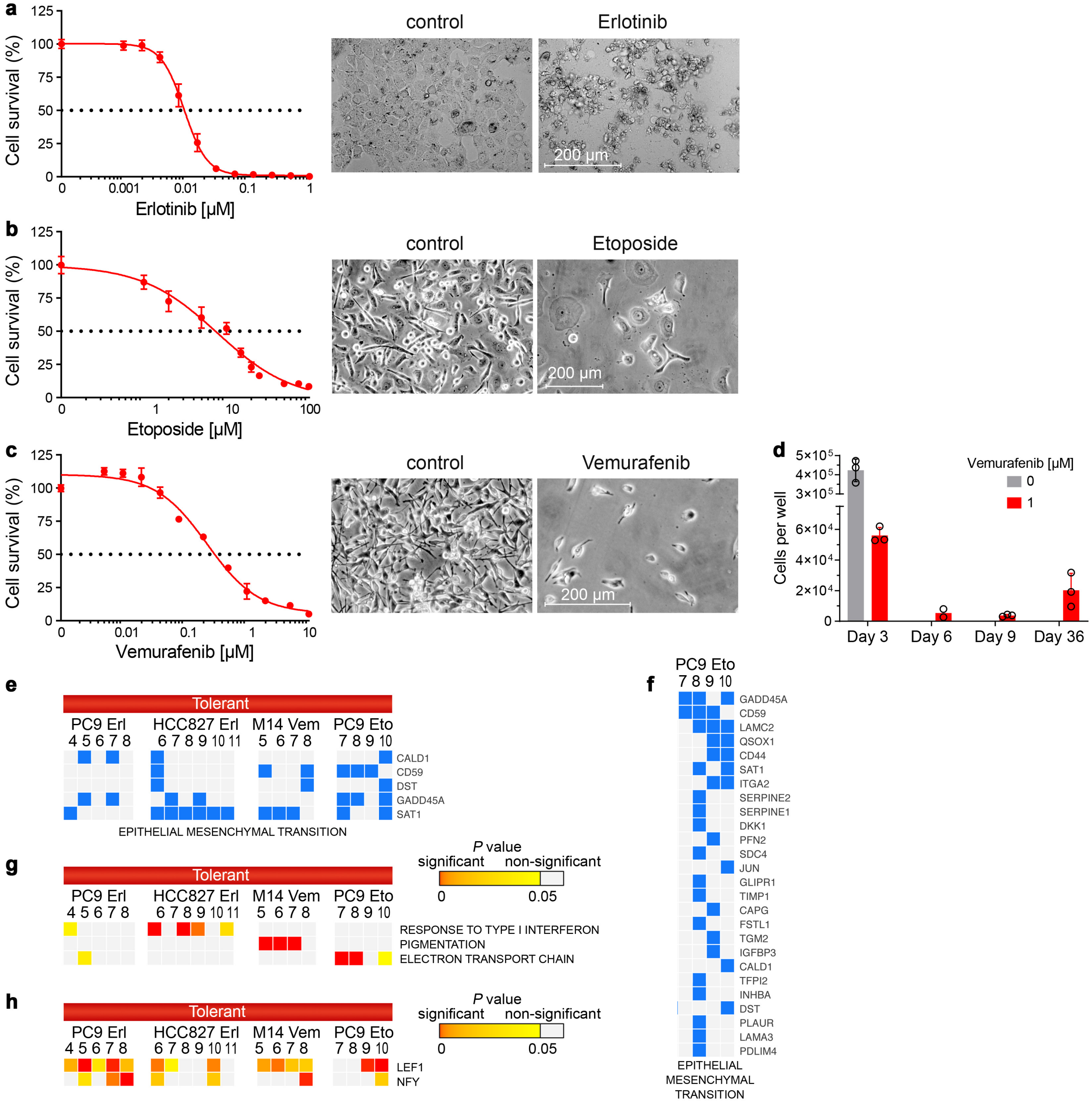

Supplementary Fig. 6. Cell line- and treatment-specific responses. a, Dose response of HCC827 cells to erlotinib compared to DMSO at day 3 of treatment. **b**, Dose response of PC9 cells to etoposide compared to DMSO at day 3 of treatment using Cell Titer Glow. **c**, Dose response of melanoma M14 cells to vemurafenib compared to DMSO using Cell Titer Glow. Dose response data in (a-c) represents mean ± SD for n = 4 replicate wells. Representative microscopic images (20x) of cells treated for a Drop-seq experiment were obtained using Zeiss microscope in (a) and Nikon light microscope in (b) and (c). Images were taken of the

cells prepared in parallel to scRNA-seq samples. **d**, Long-term survival assays of M14 cells treated with vemurafenib (1 μM). 8.1×10^4 cells were seeded per well and cell counts were performed using a hemocytometer on indicated days. Data is presented as mean ± SD for *n* = 3 replicates, but for Day 6 as a mean value for *n* = 2 replicates. **e**, Common markers associated with EMT. Shown are the markers appearing in at least three out of four samples. **f**, PC9 cell markers of etoposide-tolerant clusters associated with EMT. **g**, Enrichment analysis for gene relations to GO BP terms or KEGG pathways (MSigDB Collections) is shown for top markers of tolerant clusters ($P < 0.05$). Terms appearing highly significant in at least in three out of four different treatments are shown. Clusters of tolerant cells are indicated with a red box. **h**, Enrichment analysis of the top cluster markers for genes with occurrence of transcription factor binding sites in the regions spanning up to 4 kb around their transcription start sites (TFT). TFTs commonly enriched among the markers of DT clusters in four different cell models are shown. In (g) and (h), data represents right tail *P* values, two-sided binomial statistical test, adjusted for multiple testing using Benjamini-Hochberg FDR method.

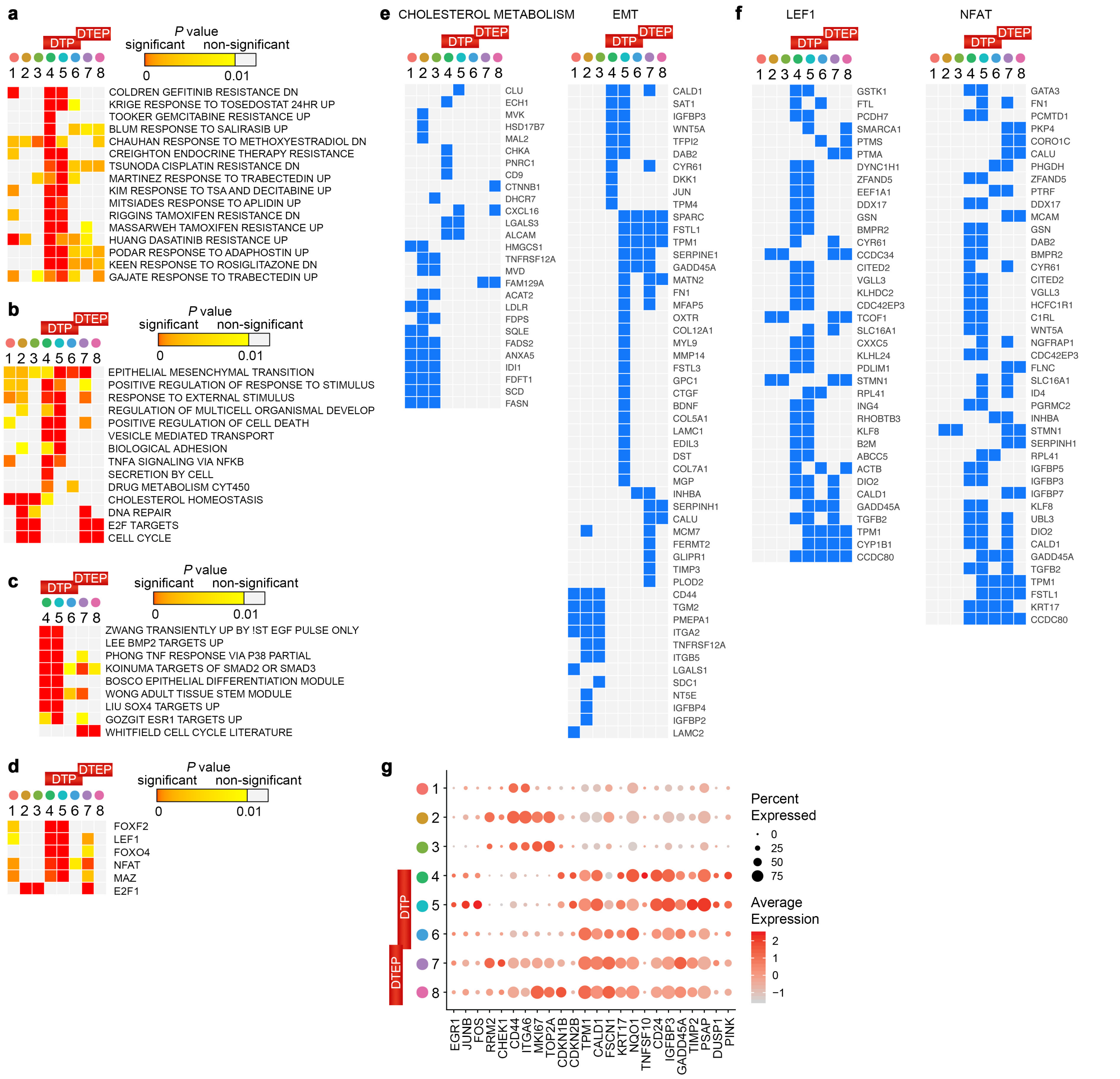

Supplementary Fig. 7. Drop-seq of consecutive samples reveals biological processes and pathways associated with drug tolerance. a, Enrichment analysis for gene sets associated with chemical and genetic perturbations (CGP, MSigDB Collections) reveals that the top cluster markers (*P* < 0.05) were previously identified to be upregulated in response to various treatments, including those associated with resistance. Consistent with changes associated with EGFR TKI resistance, the top signature is COLDREN_GEFITINIB_RESISTANCE_DN, representing genes upregulated in cell lines highly sensitive to

gefitinib. **b**, Enrichment analysis for genes in relation to GO BP terms or hallmark gene sets (MSigDB Collections) is shown for top cluster markers. Data is shown for gene sets with enrichment of $P \leq 10^{-6}$ distinguishing at least one tolerant state under the condition that all clusters of untreated cells would have $P > 10^{-4}$. The KEGG pathway term “DRUG METABOLISM_CYTOCHROME_P450” and four terms enriched in untreated cells are outside this range but still shown. **c**, Enrichment analysis for additional gene sets associated with chemical and genetic perturbations. Multiple gene sets associated with activation of ErbB receptors were condensed to one. In (a) and (c), gene sets overlapping with at least 9 markers in one tolerant cluster, $P < 10^{-7}$, were included. **d**, Top transcription factors for which binding sites (TFT) are enriched among the DT markers. TFs with P values < 10^{-5} at least in one cluster are listed. (a-d), data represents right tail P values, two-sided binomial statistical test, adjusted for multiple testing using Benjamini-Hochberg FDR method. **e**, Top markers belonging to hallmarks CHOLESTEROL HOMEOSTASIS and EPITHELIAL MESENCHYMAL TRANSITION (EMT) are shown for each cluster. **f**, Top markers with occurrence of transcription factor binding sites for LEF1 and NFAT from (d). **g**, Dot plot of expression for selected markers.

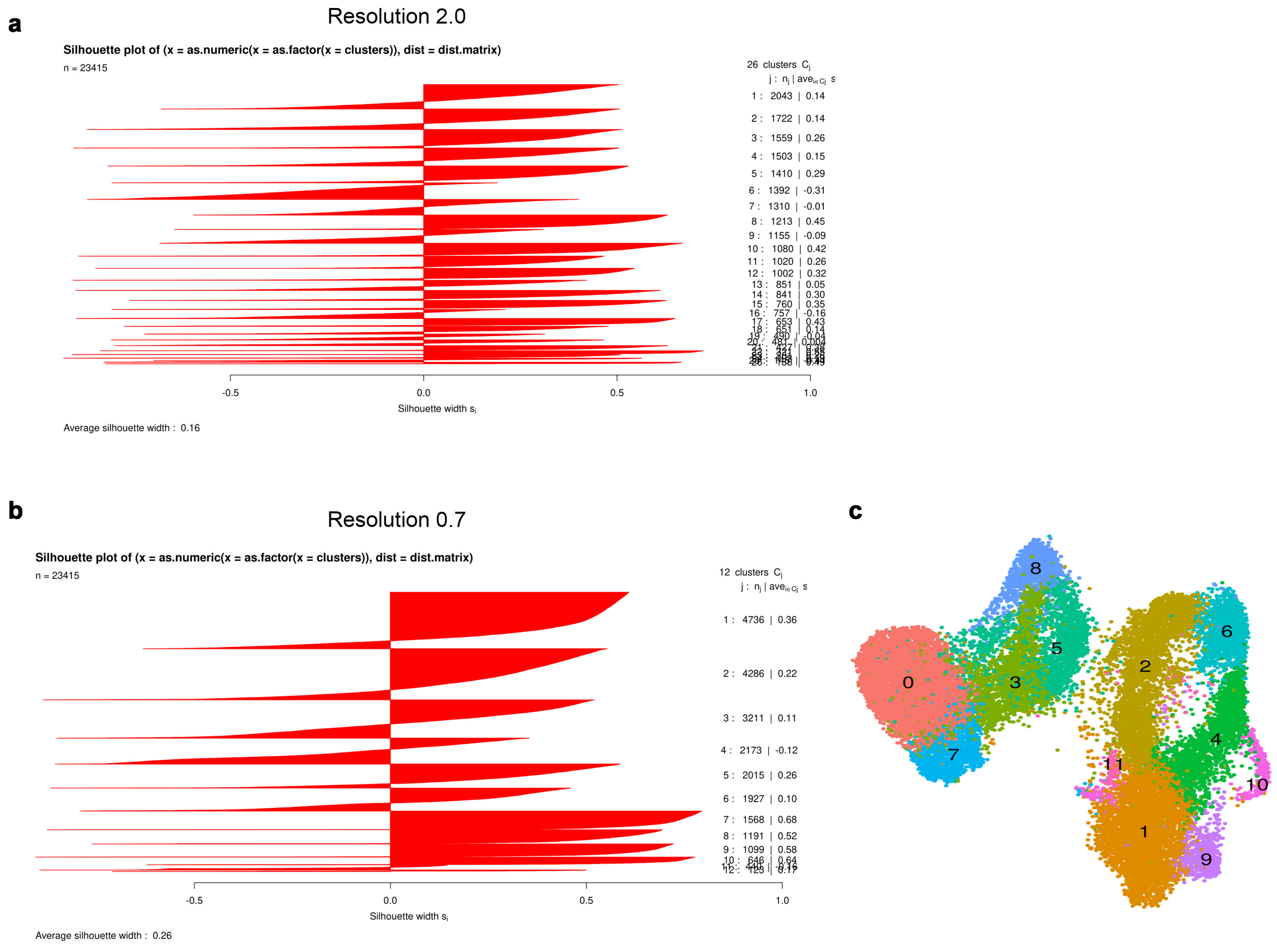

Supplementary Fig. 8. Silhouette analysis of clustering performed to distinguish DT cell populations. a, Silhouette widths calculated for each PC9 cell cluster in Fig. 4a and the resulting average silhouette width. The applied resolution was 2. **b** and **c**, Silhouette widths and UMAP representation of two PC9 samples, untreated or treated with erlotinib for 3 days, that were analyzed by 10x Genomics scRNA-seq, colored by clusters using resolution 0.7. The average silhouette width obtained with the resolution 2 is relatively low due to the negative value of silhouette width for Cluster 5, which equals to -0.31. However, low negative values appear for the relevant clusters in the clustering results generated even with the lowest resolutions, such as Cluster 3 (-0.12) for resolution 0.7 in (b). In contrast, other cells appear to form the largest clusters properly at the resolution 2. In (a) and (b), silhouette width results are reported from Cluster 0 as “1:” down.

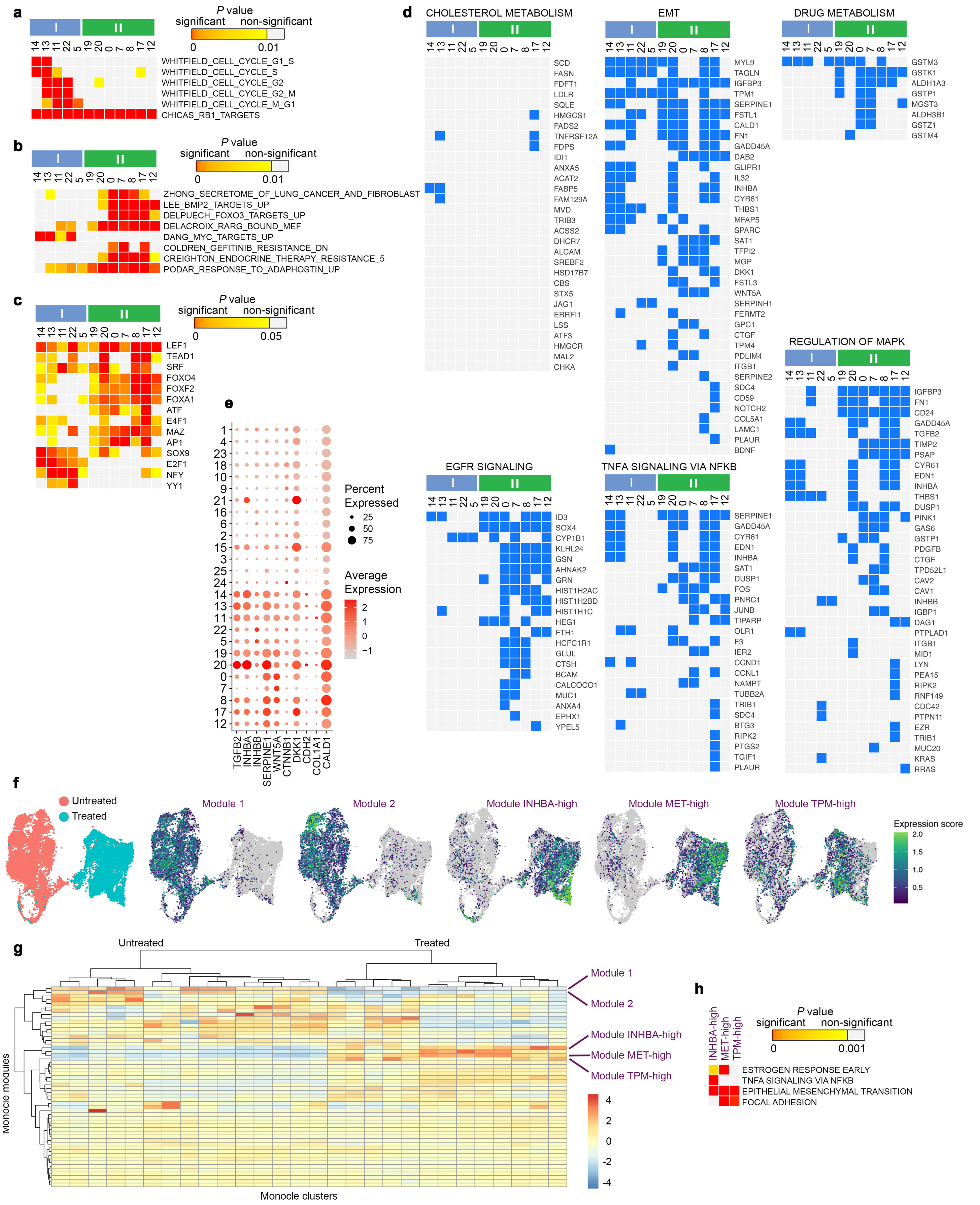
a
b
c
d
e
f
g
h
P value
significant non-significant
WHITFIELD_CELL_CYCLE_G1_S
WHITFIELD_CELL_CYCLE_S
WHITFIELD_CELL_CYCLE_G2
WHITFIELD_CELL_CYCLE_G2_M
WHITFIELD_CELL_CYCLE_M_G1
CHICAS_RB1_TARGETS
ZHONG_SECRETOME_OF_LUNG_CANCER_AND_FIBROBLAST
LEE_BMP2_TARGETS_UP
DELPUECH_FOXO3_TARGETS_UP
DELACROIX_RARG_BOUND_MEF
DANG_MYC_TARGETS_UP
COLDREN_GEFITINIB_RESISTANCE_DN
CREIGHTON_ENDOCRINE_THERAPY_RESISTANCE_5
PODAR_RESPONSE_TO_ADAPHOSTIN_UP
CHOLESTEROL METABOLISM
EMT
DRUG METABOLISM
EGFR SIGNALING
TNFA SIGNALING VIA NFKB
REGULATION OF MAPK
Percent Expressed
Average Expression
Untreated
Treated
Module 1
Module 2
Module INHBA-high
Module MET-high
Module TPM-high
Expression score
Monocle modules
Monocle clusters
ESTROGEN RESPONSE EARLY
TNFA SIGNALING VIA NFKB
EPITHELIAL MESENCHYMAL TRANSITION
FOCAL ADHESION

Supplementary Fig. 9. Prominent gene expression modules identified by enrichment analysis and Monocle reflect heterogeneity of drug tolerant cells grown in cell culture. a, Enrichment analysis for top cluster markers (P < 0.05) shows overrepresentation of CGPs related to the RB pathway. Two subpopulations of tolerant cells, I and II, are delineated by blue and green boxes. **b**, Additional CGP signatures from the enrichment analysis for top cluster markers in Fig. 4c. **c**, Enrichment analysis of top cluster markers (P < 0.05) for transcription factor targets (TFTs). Shown are LEF1, TEAD1, SRF, FOXO4, FOXF2, FOXA1, ATF, and E4F1 that had >10^3 higher P values in any of the untreated clusters, except for Cluster 21 that had lower quality cells, when compared to the majority of DT clusters. MAZ, AP1, SOX9 ,E2F1, NFY, and YY1 had >10^3 higher P values in any of the DT clusters in population II, when compared to the majority of untreated cell clusters or population I clusters. **d**, Top markers that belong to the enriched terms for hallmarks CHOLESTEROL HOMEOSTASIS and EPITHELIAL MESENCHYMAL TRANSITION (EMT), KEGG pathway DRUG METABOLISM CYTOCHROME P450, which were identified in this study, and expected signatures of CGP KOBAYASHI EGFR SIGNALING 24HR, the hallmark TNFA SIGNALING VIA NFKB, and GO REGULATION OF MAPK SIGNALING. **e**, Dot plot of expression for selected markers. **f**, Feature plots projected on UMAPs representing expression of the top two modules that were upregulated in untreated cells and the top three modules distinguishing treated cells. Clustering of untreated and erlotinib-treated for 3 days PC9 cells in a Monocle-based UMAP is shown on the *left*. **g**, Heatmap of modules of co-regulated genes, showing their differential expression in clusters of treated and untreated cells. **h**, Enrichment analysis of three EMT modules in relation to selected hallmark and KEGG gene sets. (a-c and h), data represents right tail P values, two-sided binomial statistical test, adjusted for multiple testing using Benjamini-Hochberg FDR method.

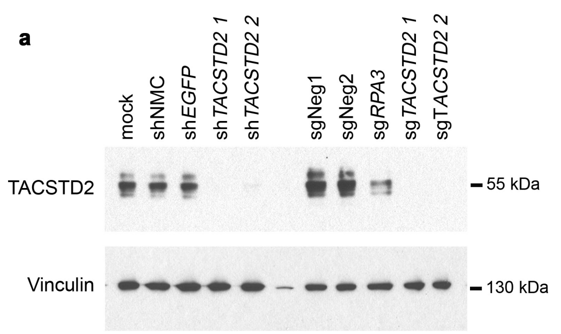

b

Sample	Reads	% Unmodified	% NHEJ
NEGATIVE CONTROL sgRPA3	121398	99.50	0.50
NEGATIVE CONTROL sgSERPINE1	110854	98.20	1.80
sgRPA3	96866	4.10	95.90
sgTACSTD2 1	52666	2.80	97.20
sgTACSTD2 2	50638	3.30	96.70
sgSERPINE1	90414	10.30	89.70

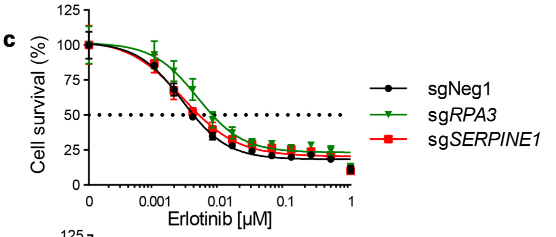

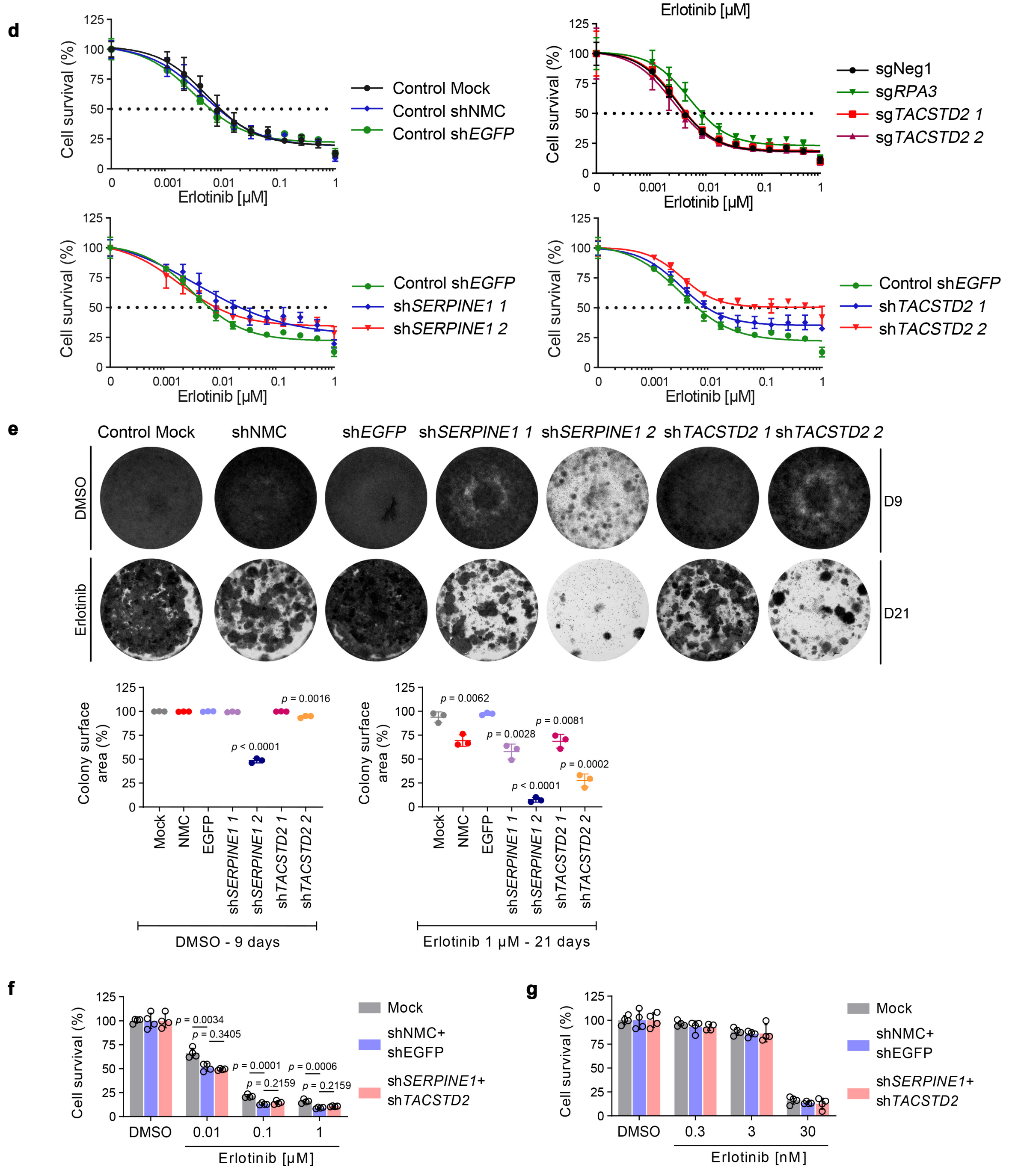

Supplementary Fig. 10. Inhibiting individual tolerant cell markers fails to eradicate drug tolerance. a, Immunoblot analysis of cell lysates prepared from PC9 cells that were transduced with indicated shRNAs and sgRNAs. Inhibition of CYP1B1 was not pursued further as transduction with shRNAs and sgRNAs was toxic to untreated cells. Controls: NMC – non-mammalian sequence, EGFP – *EGFP* sequence, Neg1 and Neg2 – non-targeting sequences. *RPA3* is an essential gene. Vinculin served as a loading control. Source data is provided as a Source Data file. The experiment was repeated 2 times, which gave similar results. **b**, Percentage of mutated allele in cells transduced with sgRNA-encoding lentiviruses. **c**, Dose response to erlotinib compared to DMSO in cells transduced with either Negative Control 1 sgRNA (sgNeg1), or with CRISPR knockouts of the essential gene *RPA3*, markers of resistance *SERPINE1* and *TACSTD2* (1 and 2 are two different sgRNAs for *TACSTD2*). **d**, Dose response to erlotinib compared to DMSO in cells transduced with *SERPINE1* or *TACSTD2* shRNAs. The effect of lentiviral expression of shRNA to green fluorescent protein (shEGFP) or Non-Mammalian Control shRNA (shNMC) is shown as a control. In (c) and (d), treatments with erlotinib and DMSO proceeded for 3 days and Hoechst staining was performed to determine relative numbers of cells. Mean ± SD for $n = 4$ replicate wells. **e**, PC9 cells were transduced as in (a) and (b) followed by DMSO for 9 days or erlotinib (1 μM) for 21 days with media/drug changes every 3 days. Surviving cells were stained with crystal violet, photographed using Azure System, and plate colony surface area was quantified using ZEN 2.6.. Data represents mean ± SD for $n = 3$ replicate wells. (**f** and **g**) Cell survival assays by SYTO83 of the double knockdown in PC9 (f) and HCC827 (g). Mean ± SD for $n = 4$ replicate wells. In e-g, two-tailed P values were determined by unpaired t test relative to the control mock via GraphPad Prism 7.

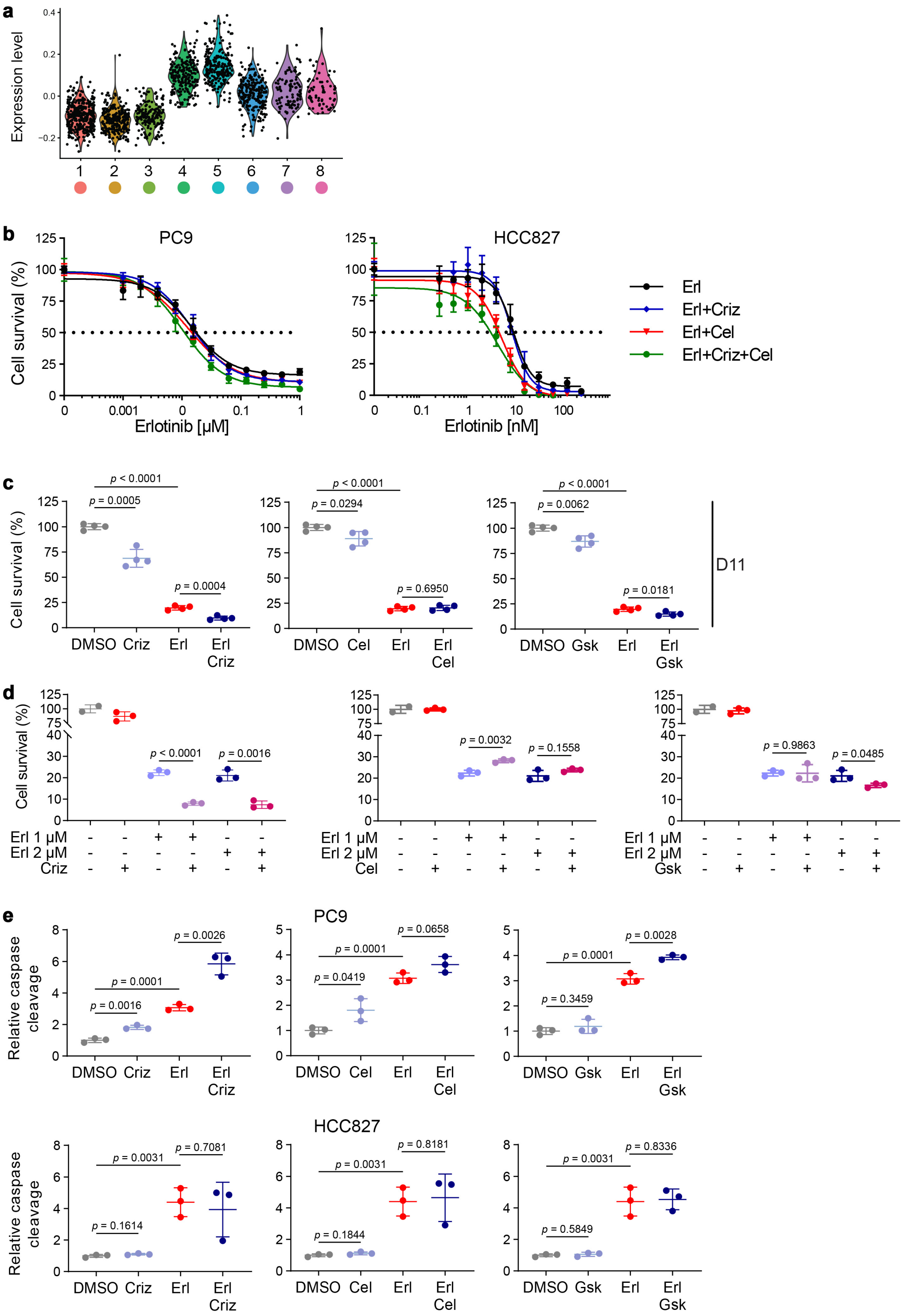
a
Expression level
1 2 3 4 5 6 7 8
b
PC9
HCC827
Cell survival (%)
Erlotinib [μM]
Erlotinib [nM]
Erl
Erl+Criz
Erl+Cel
Erl+Criz+Cel
c
p < 0.0001
p = 0.0005
p = 0.0004
p = 0.0294
p = 0.6950
p = 0.0062
p = 0.0181
D11
DMSO Criz Erl Erl Criz
DMSO Cel Erl Erl Cel
DMSO Gsk Erl Erl Gsk
d
p < 0.0001
p = 0.0016
p = 0.0032
p = 0.1558
p = 0.9863
p = 0.0485
Erl 1 μM
Erl 2 μM
Criz
Cel
Gsk
e
PC9
Relative caspase cleavage
p = 0.0026
p = 0.0001
p = 0.0016
p = 0.0658
p = 0.0419
p = 0.0028
p = 0.3459
HCC827
p = 0.0031
p = 0.7081
p = 0.1614
p = 0.8181
p = 0.1844
p = 0.8336
p = 0.5849

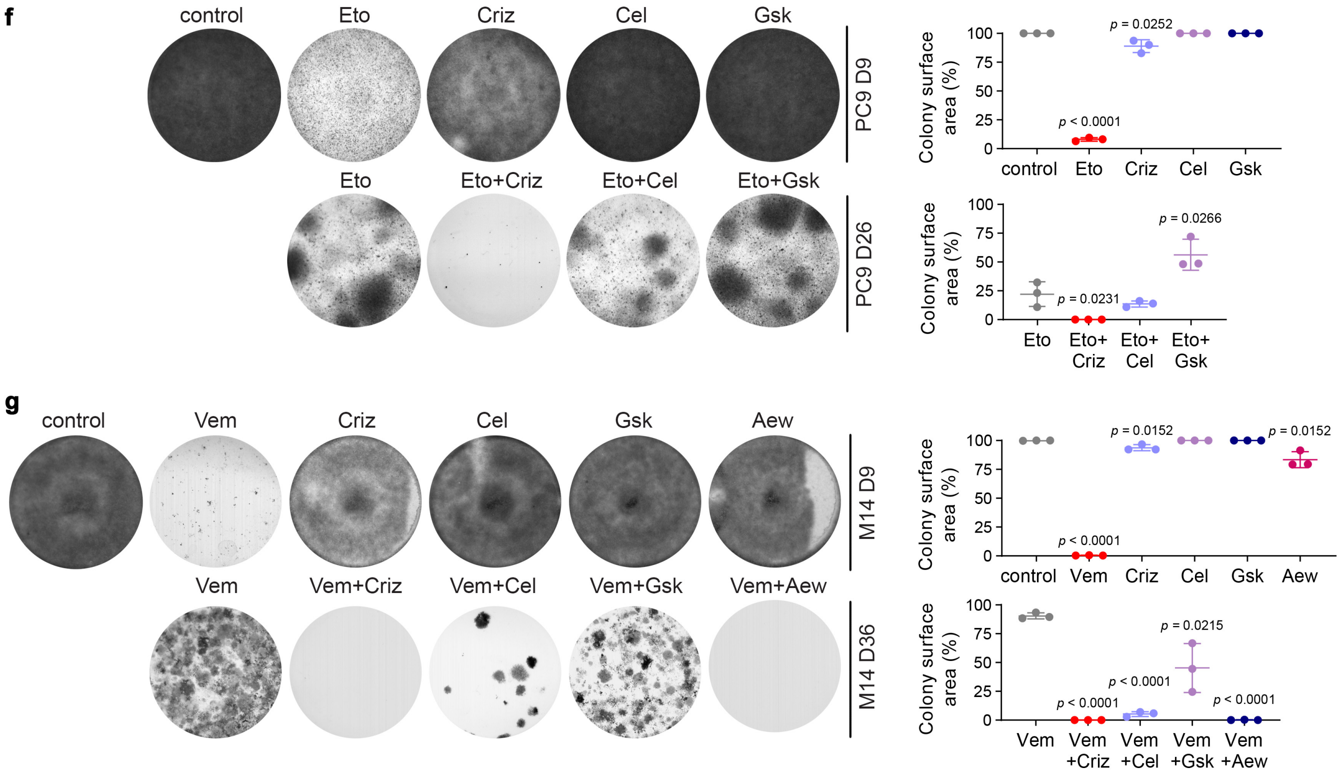

Supplementary Fig. 11. The combination of erlotinib with crizotinib and celastrol was effective in blocking proliferation of tolerant cells. a, Violin plots of DT state markers, which are predicted to be affected by crizotinib, are shown among PC9 cell clusters from Fig. 1e. The violin plot was generated with the same data as crizotinib UMAP in Fig. 5c. **b**, Dose response of PC9 and HCC827 cells treated for 3 days with erlotinib as a single agent and in combination. **c**, Survival assays of PC9 cells treated for 11 days with erlotinib as a single agent and in combination. **d**, Survival assays of PC9 treated for 3 days with two concentrations of erlotinib in combination with three other drugs as measured by Cell Titer Glo. **e**, Apoptosis assessment by caspase cleavage assay on drug-treated PC9 and HCC827 cells. In (b) through (e), mean ± SD for $n = 4$ replicate wells. **f** and **g**, Colony formation assays of PC9 and M14 cells, respectively, treated for indicated number of days with Eto (etoposide) or Vem (vemurafenib) and other drugs. Celastrol was used at 1 µM concentration. Representative crystal violet stainings are shown. Plate colony surface area is shown as mean ± SD for $n = 3$ replicate wells. In (c) through (g), two-tailed p values were determined by unpaired t test relative to the control or targeted drug via GraphPad Prism 7. The drug concentrations are as in Fig. 5f unless noted otherwise.

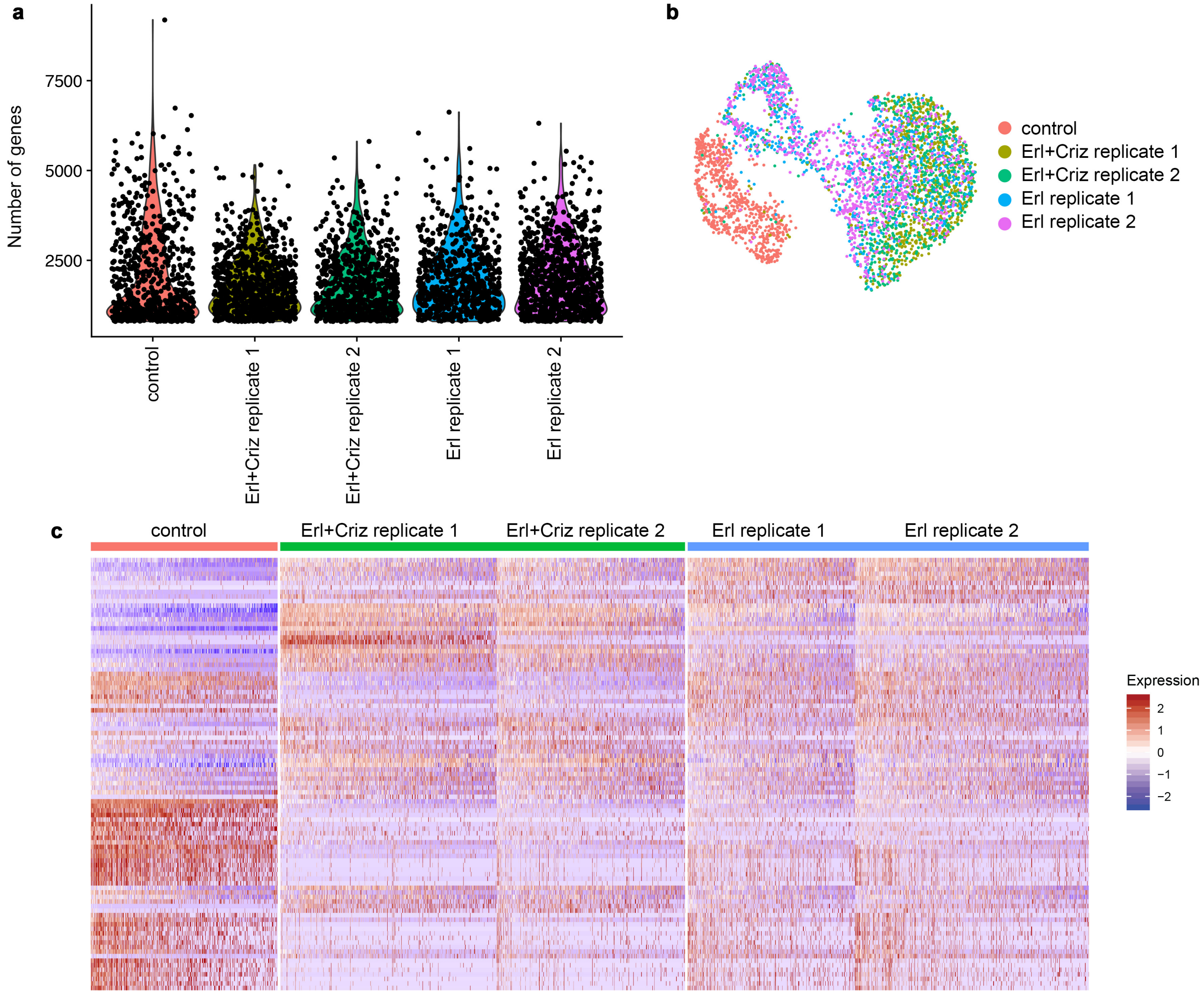

Supplementary Fig. 12. Independent replicates of drug tolerant cells generate highly reproducible Drop-seq data. a, Distribution of the number of sequenced genes per cell, per sample treated with erlotinib alone, in combination with crizotinib, and untreated. Two biological replicates were performed to exclude potential batch effects. **b**, UMAP representation of the samples. **c**, Heatmap of top markers distinguishing the differently treated cells and their subpopulations.

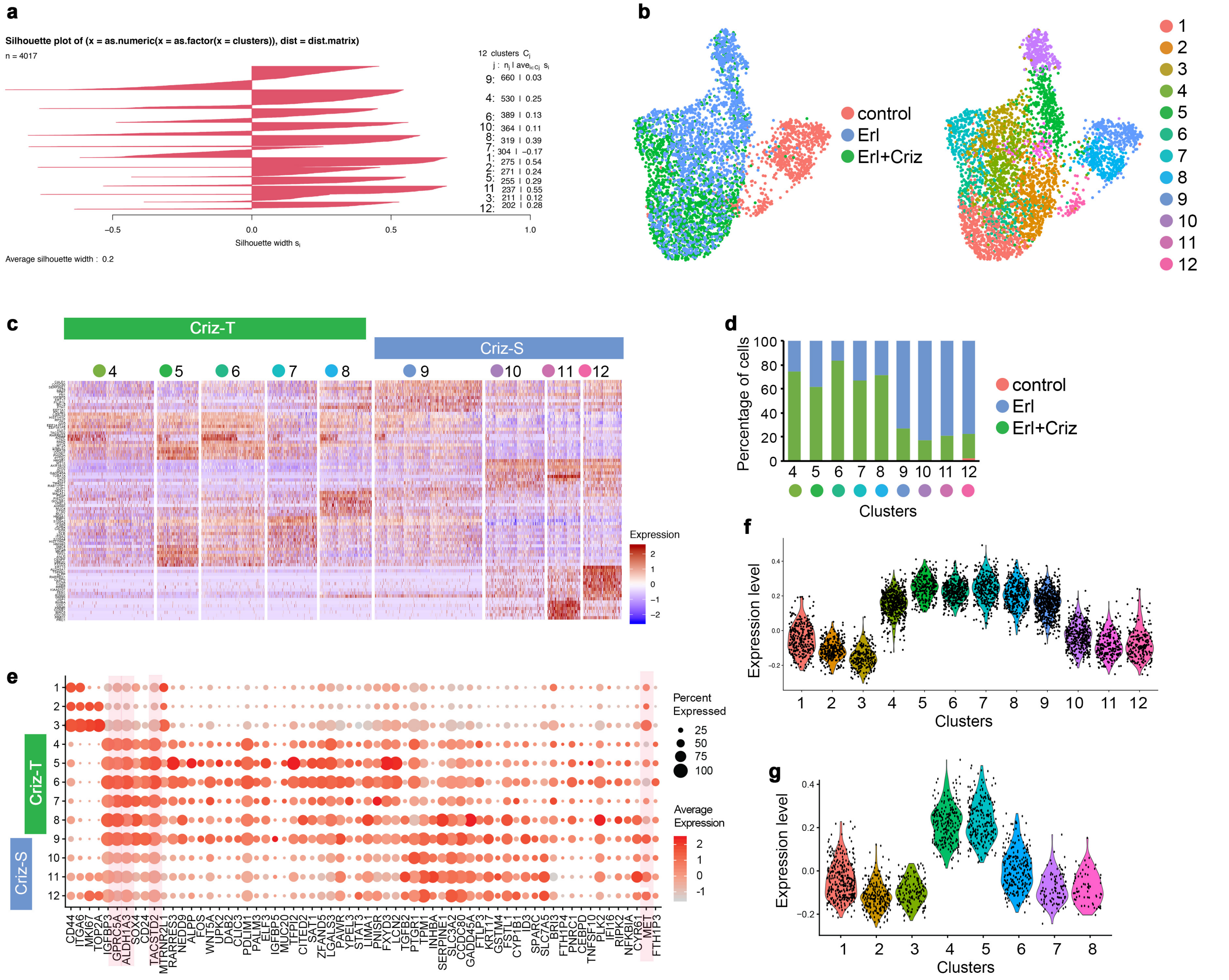

Supplementary Fig. 13. Exploring the combination of erlotinib and crizotinib for targeting distinctive molecular signatures. a, Silhouette widths calculated for each cluster in Fig. 6b and the resulting average silhouette width. **b**, Regressing cell cycle genes does not change overall attribution of DT states. UMAP representation of PC9 cells colored by treatment (*left panel*) or clusters (*right panel*) after regressing cell cycle genes from data in Fig. 6b is shown. **c**, Heatmap of the top markers for each Criz-T and Criz-S cluster. **d**, Percentage of cells from each sample mapped by Seurat to each cluster. The distribution shows the percentage of cells with single-agent erlotinib treatment and dual-agent erlotinib and crizotinib treatment in

each tolerant cluster; no control cells were detected in tolerant clusters. **e**, Average expression levels of top cluster markers across the untreated cells (Clusters 1, 2 and 3) and treated cells (Criz-T and Criz-S Clusters from 4 to 12) are presented as dot plots. The color of each dot represents the average expression level from low (grey) to high (red), and the size of each dot represents the percentage of the cells expressing the gene. Markers of interest are highlighted in red. **f**, Violin plots of Criz-T signature in each cluster of the untreated cells and treated cells. **g**, Violin plots of Criz-T signature in each state of PC9 cells treated with erlotinib. The states of PC9 cells are from the set of consecutive samples in Fig. 1. In (f) and (g), the Criz-T signature was generated by combining markers of Criz-T clusters. Next, their overall expression level was calculated for each cell by Seurat and single-cell expression distributions were visualized for each cluster.

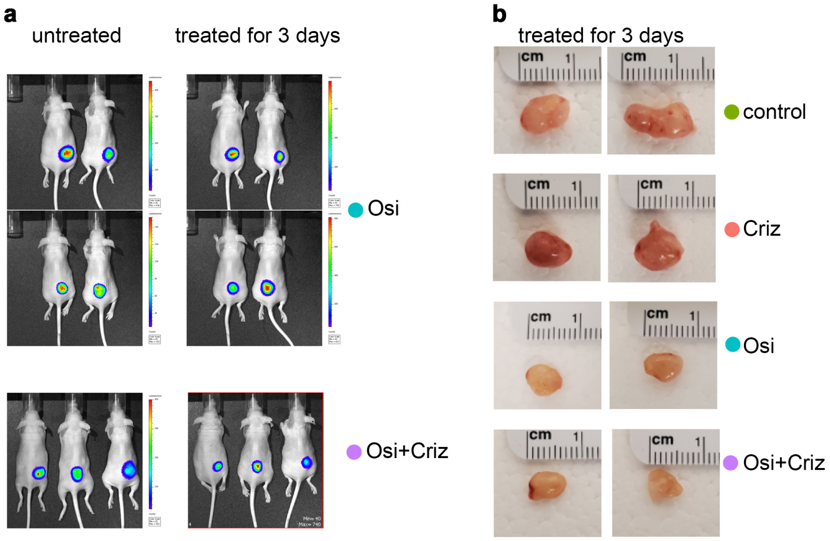
a
untreated
treated for 3 days
Osi
Osi+Criz
b
treated for 3 days
control
Criz
Osi
Osi+Criz

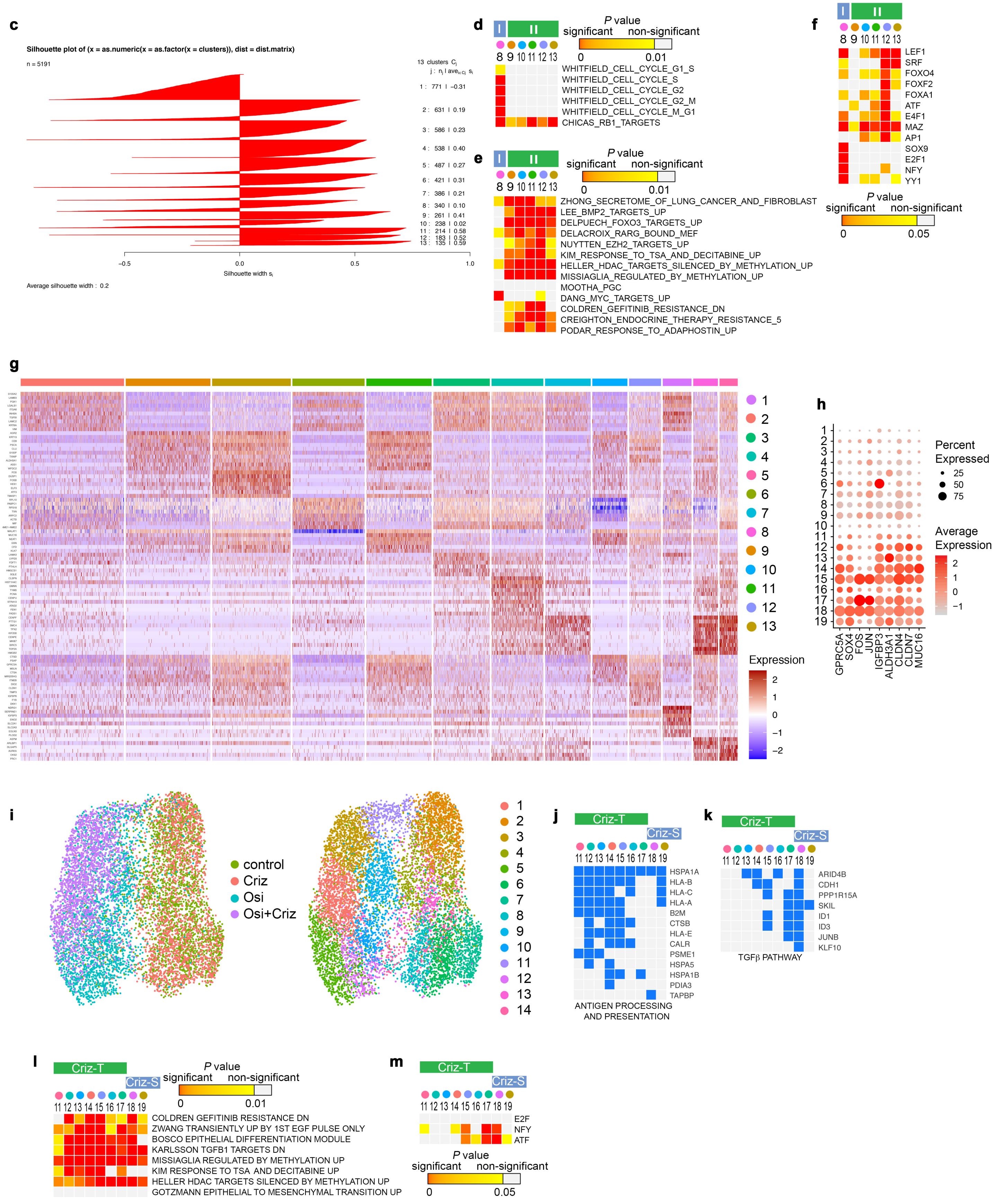
c
Silhouette plot of (x = as.numeric(x = as.factor(x = clusters)), dist = dist.matrix)
n = 5191
13 clusters C_j
j : n_j | $ave_{i \in Cj}$ s_i
1 : 771 | −0.31
2 : 631 | 0.19
3 : 586 | 0.23
4 : 538 | 0.40
5 : 487 | 0.27
6 : 421 | 0.31
7 : 386 | 0.21
8 : 340 | 0.10
9 : 261 | 0.41
10 : 238 | 0.02
11 : 214 | 0.58
12 : 183 | 0.52
13 : 135 | 0.59
Silhouette width s_i
Average silhouette width : 0.2
d
I
II
8 9 10 11 12 13
P value
significant non-significant
0 0.01
WHITFIELD_CELL_CYCLE_G1_S
WHITFIELD_CELL_CYCLE_S
WHITFIELD_CELL_CYCLE_G2
WHITFIELD_CELL_CYCLE_G2_M
WHITFIELD_CELL_CYCLE_M_G1
CHICAS_RB1_TARGETS
e
ZHONG_SECRETOME_OF_LUNG_CANCER_AND_FIBROBLAST
LEE_BMP2_TARGETS_UP
DELPUECH_FOXO3_TARGETS_UP
DELACROIX_RARG_BOUND_MEF
NUYTTEN_EZH2_TARGETS_UP
KIM_RESPONSE_TO_TSA_AND_DECITABINE_UP
HELLER_HDAC_TARGETS_SILENCED_BY_METHYLATION_UP
MISSIAGLIA_REGULATED_BY_METHYLATION_UP
MOOTHA_PGC
DANG_MYC_TARGETS_UP
COLDREN_GEFITINIB_RESISTANCE_DN
CREIGHTON_ENDOCRINE_THERAPY_RESISTANCE_5
PODAR_RESPONSE_TO_ADAPHOSTIN_UP
f
LEF1
SRF
FOXO4
FOXF2
FOXA1
ATF
E4F1
MAZ
AP1
SOX9
E2F1
NFY
YY1
P value
significant non-significant
0 0.05
g
Expression
h
Percent Expressed
Average Expression
GPRC5A
SOX4
FOS
JUN
IGFBP3
ALDH3A1
CLDN4
CLDN7
MUC16
i
control
Criz
Osi
Osi+Criz
j
Criz-T
Criz-S
11 12 13 14 15 16 17 18 19
HSPA1A
HLA-B
HLA-C
HLA-A
B2M
CTSB
HLA-E
CALR
PSME1
HSPA5
HSPA1B
PDIA3
TAPBP
ANTIGEN PROCESSING AND PRESENTATION
k
ARID4B
CDH1
PPP1R15A
SKIL
ID1
ID3
JUNB
KLF10
TGFβ PATHWAY
l
COLDREN GEFITINIB RESISTANCE DN
ZWANG TRANSIENTLY UP BY 1ST EGF PULSE ONLY
BOSCO EPITHELIAL DIFFERENTIATION MODULE
KARLSSON TGFB1 TARGETS DN
MISSIAGLIA REGULATED BY METHYLATION UP
KIM RESPONSE TO TSA AND DECITABINE UP
HELLER HDAC TARGETS SILENCED BY METHYLATION UP
GOTZMANN EPITHELIAL TO MESENCHYMAL TRANSITION UP
m
E2F
NFY
ATF

Supplementary Fig. 14. Enrichment analysis reflect heterogeneity of drug tolerant cells in xenograft model. a, Images of representative mice with established PC9 tumors (untreated) and the same mice after 3 days of treatments with osimertinib or osimertinib and crizotinib. **b**, Tumors resected from mice receiving osimertinib and crizotinib treatments for 3 days compared to vehicle treatment. These tumors were used for Drop-seq experiments. **c**, Silhouette widths calculated for each cluster in xenografts treated with osimertinib or vehicle in Fig. 7a and the resulting average silhouette width. Besides Cluster 2 (-0.31), which is separated in two distinctive subpopulations in Fig. 7a, all other large clusters give good silhouette width. **d**, Enrichment analysis shows overrepresentation of CGP gene sets related to the RB pathway. **e**, Additional CGP signatures from the enrichment analysis for top cluster markers in Fig. 7c. The terms with more than 7 markers per cluster, $P < 10^{-7}$ in at least in one cluster, and > 10^{-3} difference in any of the clusters of another treatment were included. **f**, Enrichment analysis for TFTs. **g**, Heatmap of top markers for each cluster. Cluster annotation as in Fig. 7a. **h**, Dot plot of expression for selected markers. The clusters 1 through 19 are from Fig. 7c. **i**, Regressing cell cycle genes does not change overall attribution of DT populations. UMAP representation of xenograft tumor cells colored by treatment (*left panel*) or clusters (*right panel*) after regressing cell cycle genes. **j**, Top markers that belong to the enriched GO BP term ANTIGEN PROCESSING AND PRESENTATION, which are either crizotinib-tolerant (Criz-T) or crizotinib-sensitive (Criz-S). **k**, Top markers that belong to the enriched KEGG pathway TGF BETA SIGNALING. **l**, Enrichment analysis for selected CGP gene sets. **m**, Enrichment analysis for binding sites (TFT) of transcription factors that were previously identified in the PC9 cell culture experiment in Fig. 6f. In (d-f), two subpopulations of tolerant cells, I and II, are delineated by blue and green boxes. In (j-m), Criz-T clusters and Criz-S clusters are shown by green and blue box, respectively. In (d-f), (l) and (m), enrichment analysis was performed using top cluster markers ($P < 0.05$); data represents right tail P values, two-sided binomial statistical test, adjusted for multiple testing using Benjamini-Hochberg FDR method.

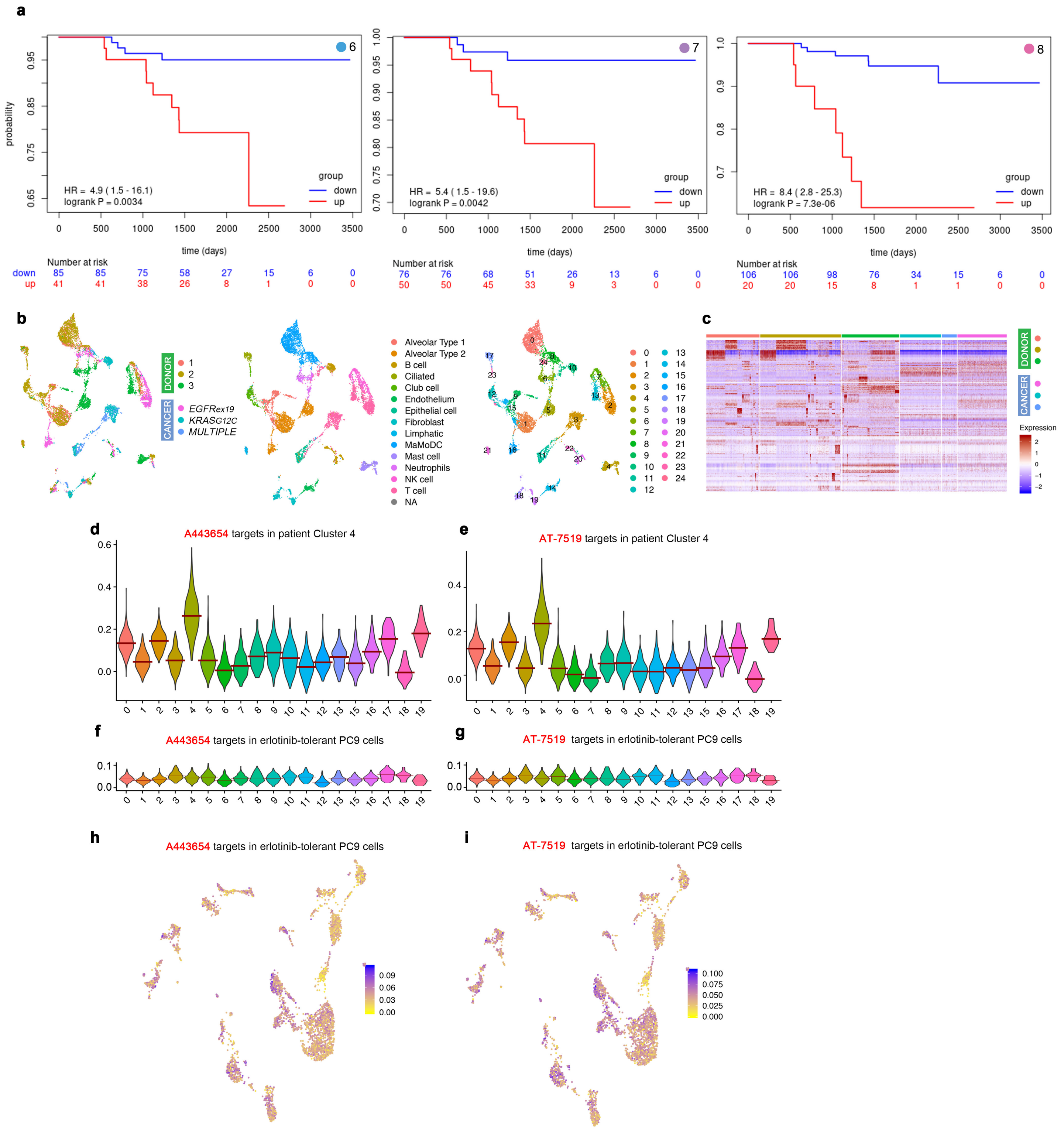

Supplementary Fig. 15. Patient survival and LINCS analysis of EGFR-mutant and KRASG12C patient tumors. **a**, Kaplan-Meier estimates of survival before death/censored in EGFR-mutant lung adenocarcinomas (as in Fig. 8a) with significantly upregulated markers of individual DT states 6, 7 or 8 compared to patients, where the same markers showed decreased expression or no significant change ($P > 0.05$). Univariate Cox

regression was used to determine Hazard Ratio (HR) and log rank *P* values. **b**, UMAP representation of cells from three donors and three NSCLC patients colored by cell types. *EGFRex19* – tumor with *EGFR*ex19 mutation; *KRASG12C* - tumor with *KRAS*G12C mutation; *MULTIPLE* – multiple oncogenic driver mutations have been identified in this tumor. MaMoDC – macrophages, monocytes and dendritic cells combined; NA – failed to be classified; others as indicated. As anticipated, the tissue composition of independently collected samples varied. Despite deficiency in populations in some samples, both donor and patient samples contributed to a majority of cell populations. UMAP positioning of several cell types varied between donors and patients, and between different patients. In particular, fibroblasts and endothelial cells from tumor samples clustered uniquely. **c**, Heatmap showing the expression of the top markers distinguishing the 25 identified clusters for each donor and patient in (b). **d**, Violin plot showing expression level of genes targeted by the AKT inhibitor A443654 identified in the LINCS analysis. **e**, Violin plot showing expression level of genes targeted by the CDK inhibitor AT-7519 identified in the LINCS analysis. **f**, Violin plot showing expression level of genes targeted by the AKT inhibitor A443654 identified in the LINCS analysis. **g**, Violin plot showing expression level of genes targeted by the CDK inhibitor AT-7519 identified in the LINCS analysis. **h**, Feature plot showing cells colored by expression level of genes targeted by the AKT inhibitor A443654 identified in the LINCS analysis. **i**, Feature plot showing cells colored by expression level of genes targeted by the CDK inhibitor AT-7519 identified in the LINCS analysis. The score was calculated by Seurat and was based in (d) and (e) on expression level of markers of *EGFR*ex19 patient cancer cells (Cluster 4 in Fig. 8e and genes Supplementary Data 27), and in (f-i) on expression level of markers of earlier DT states in PC9 cells (Clusters 4 and 5 in Fig. 1 and markers of Clusters 4 and 5 from Supplementary Data 1).

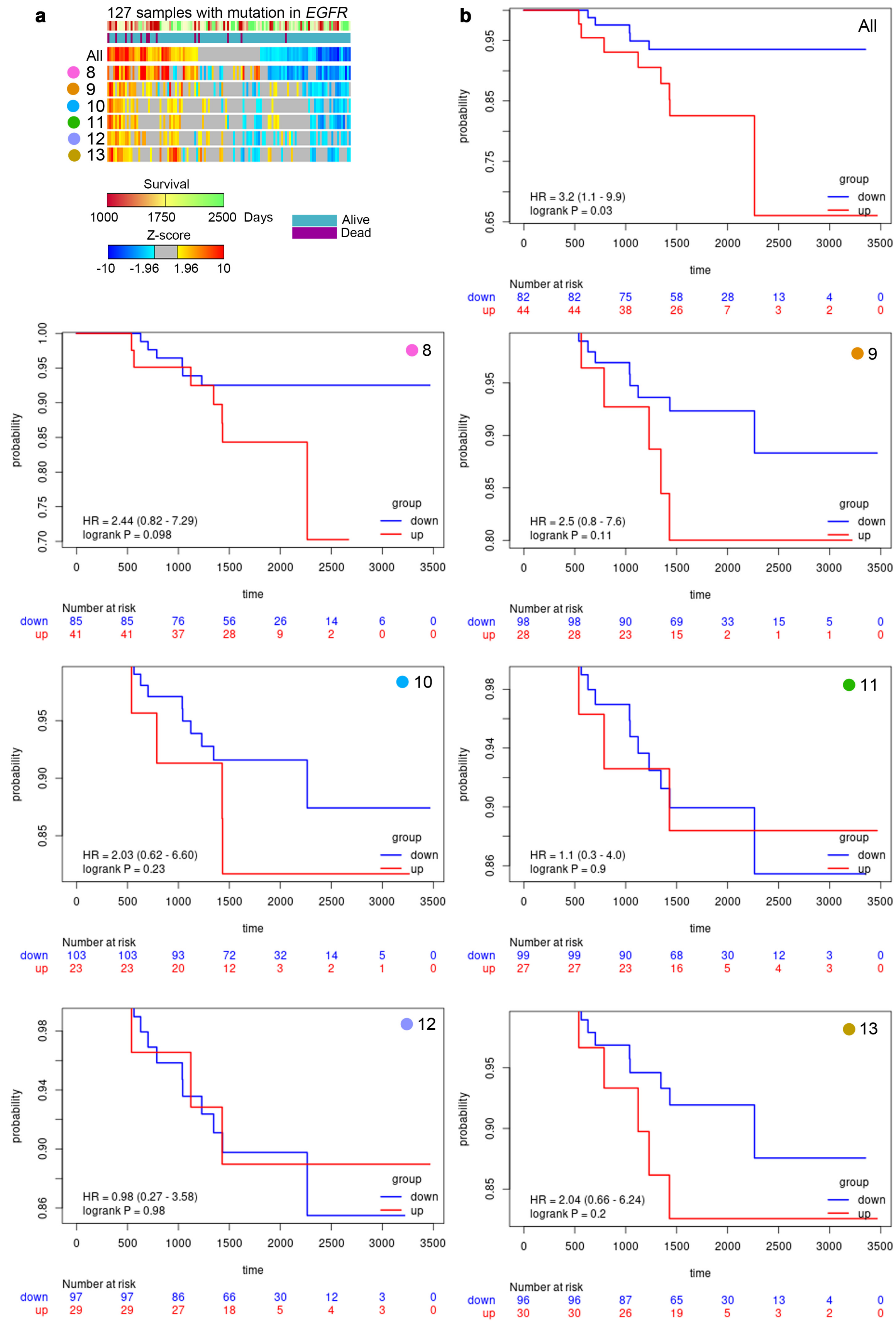
a
127 samples with mutation in *EGFR*
All
8
9
10
11
12
13
Survival
1000 1750 2500 Days
Alive
Dead
Z-score
-10 -1.96 1.96 10
b
All
HR = 3.2 (1.1 - 9.9)
logrank P = 0.03
Number at risk
down 82 82 75 58 28 13 4 0
up 44 44 38 26 7 3 2 0
8
HR = 2.44 (0.82 - 7.29)
logrank P = 0.098
Number at risk
down 85 85 76 56 26 14 6 0
up 41 41 37 28 9 2 0 0
9
HR = 2.5 (0.8 - 7.6)
logrank P = 0.11
Number at risk
down 98 98 90 69 33 15 5 0
up 28 28 23 15 2 1 1 0
10
HR = 2.03 (0.62 - 6.60)
logrank P = 0.23
Number at risk
down 103 103 93 72 32 14 5 0
up 23 23 20 12 3 2 1 0
11
HR = 1.1 (0.3 - 4.0)
logrank P = 0.9
Number at risk
down 99 99 90 68 30 12 3 0
up 27 27 23 16 5 4 3 0
12
HR = 0.98 (0.27 - 3.58)
logrank P = 0.98
Number at risk
down 97 97 86 66 30 12 3 0
up 29 29 27 18 5 4 3 0
13
HR = 2.04 (0.66 - 6.24)
logrank P = 0.2
Number at risk
down 96 96 87 65 30 13 4 0
up 30 30 26 19 5 3 2 0
probability
time
group
down
up

c

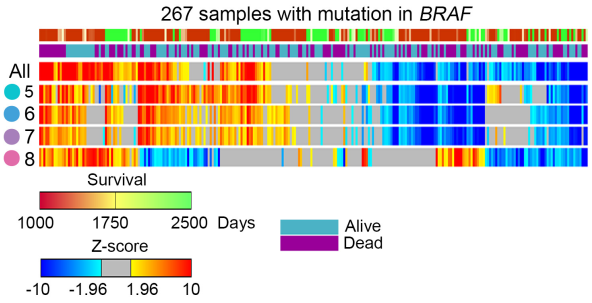
267 samples with mutation in BRAF
All
5
6
7
8
Survival
1000 1750 2500 Days
Alive
Dead
Z-score
-10 -1.96 1.96 10

d

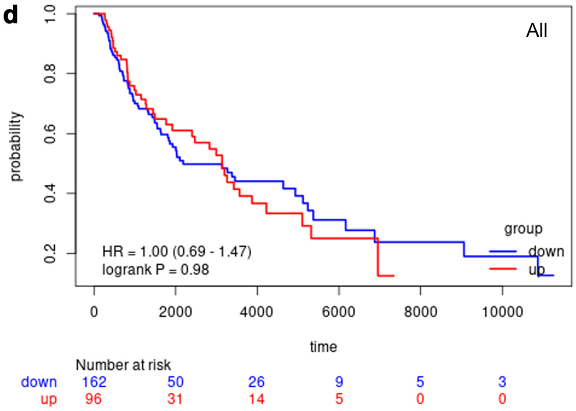
All
probability
time
group
down
up
HR = 1.00 (0.69 - 1.47)
logrank P = 0.98
Number at risk
down 162 50 26 9 5 3
up 96 31 14 5 0 0

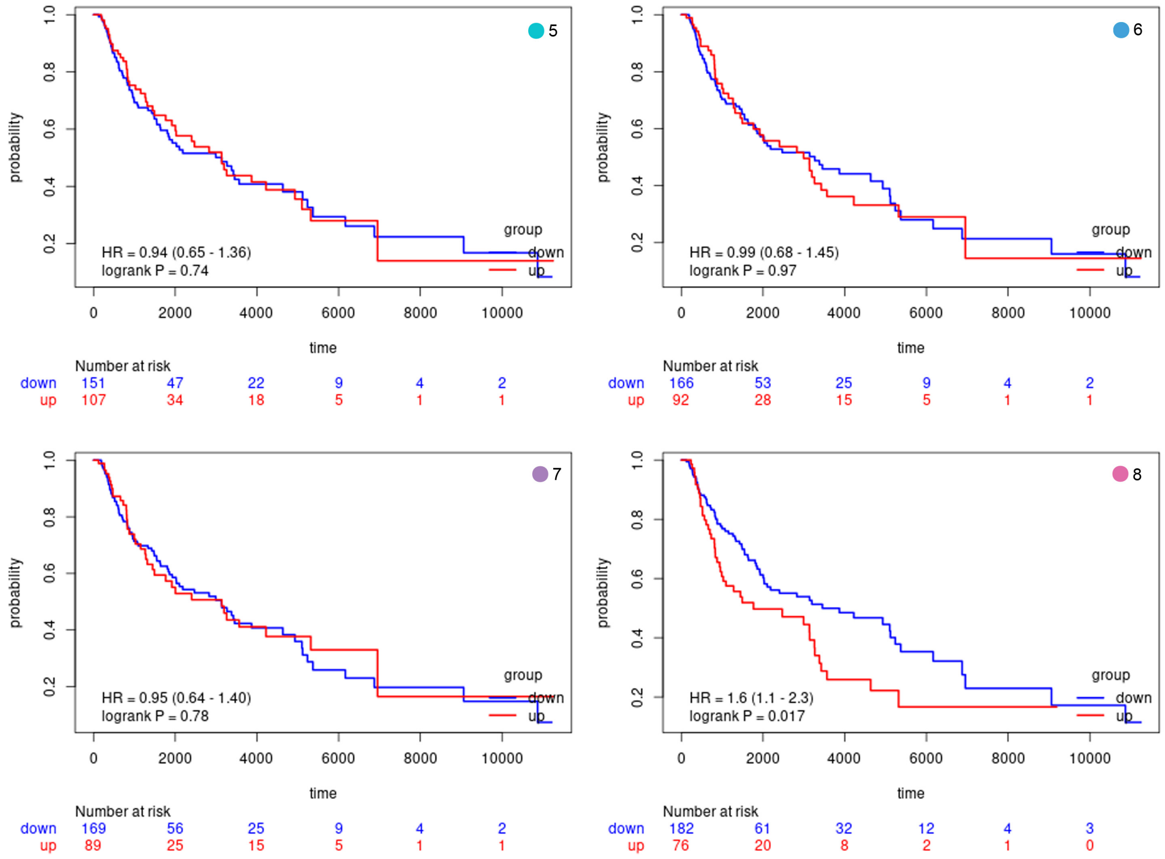
5
HR = 0.94 (0.65 - 1.36)
logrank P = 0.74
Number at risk
down 151 47 22 9 4 2
up 107 34 18 5 1 1
6
HR = 0.99 (0.68 - 1.45)
logrank P = 0.97
Number at risk
down 166 53 25 9 4 2
up 92 28 15 5 1 1
7
HR = 0.95 (0.64 - 1.40)
logrank P = 0.78
Number at risk
down 169 56 25 9 4 2
up 89 25 15 5 1 1
8
HR = 1.6 (1.1 - 2.3)
logrank P = 0.017
Number at risk
down 182 61 32 12 4 3
up 76 20 8 2 1 0

e

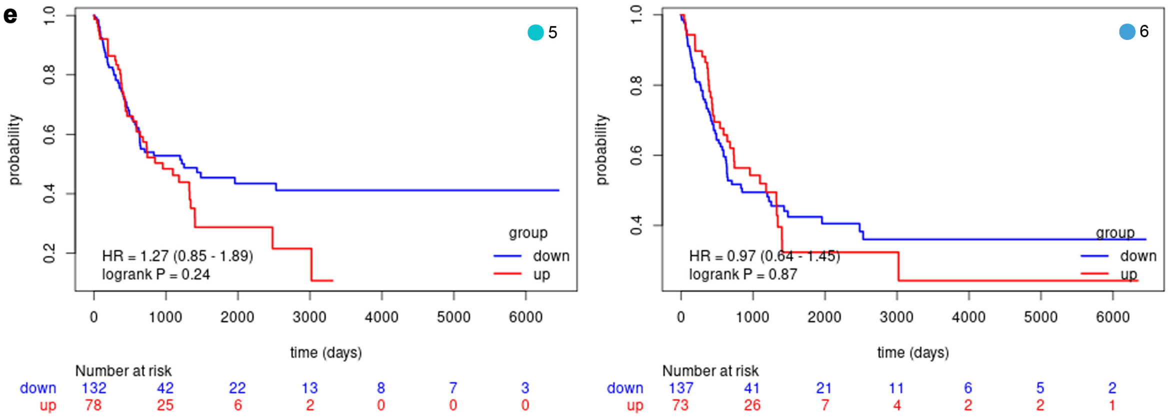
5
probability
time (days)
HR = 1.27 (0.85 - 1.89)
logrank P = 0.24
group
down
up
Number at risk
down 132 42 22 13 8 7 3
up 78 25 6 2 0 0 0
6
HR = 0.97 (0.64 - 1.45)
logrank P = 0.87
Number at risk
down 137 41 21 11 6 5 2
up 73 26 7 4 2 2 1
7
HR = 1.16 (0.77 - 1.75)
logrank P = 0.47
Number at risk
down 140 43 22 12 7 6 3
up 70 24 6 3 1 1 0
8
HR = 1.25 (0.81 - 1.93)
logrank P = 0.31
Number at risk
down 150 54 22 11 5 4 2
up 60 13 6 4 3 3 1

f

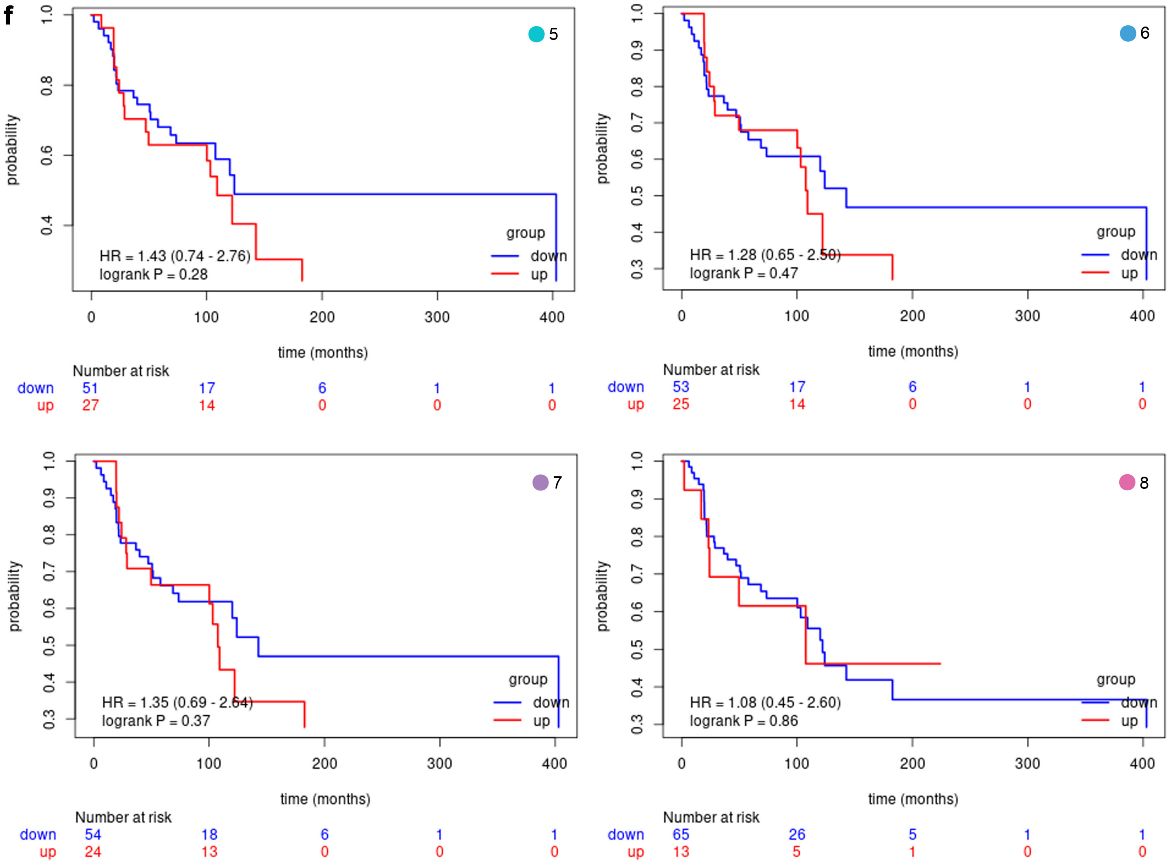
5
probability
time (months)
HR = 1.43 (0.74 - 2.76)
logrank P = 0.28
group
down
up
Number at risk
down 51 17 6 1 1
up 27 14 0 0 0
6
HR = 1.28 (0.65 - 2.50)
logrank P = 0.47
Number at risk
down 53 17 6 1 1
up 25 14 0 0 0
7
HR = 1.35 (0.69 - 2.64)
logrank P = 0.37
Number at risk
down 54 18 6 1 1
up 24 13 0 0 0
8
HR = 1.08 (0.45 - 2.60)
logrank P = 0.86
Number at risk
down 65 26 5 1 1
up 13 5 1 0 0

Supplementary Fig. 16. Patient survival analysis of EGFR-mutant NSCLCs and melanoma tumors using markers of subpopulations within drug tolerant cell population. a, Sample level enrichment analysis of DT markers, which were identified in the PC9 xenograft treated with osimertinib, was performed in EGFR-mutant lung adenocarcinomas. Expression level was determined for markers of DT clusters surviving in PC9 xenograft (Clusters 8-13 in Fig. 7a and genes in Supplementary Data 49), for all clusters together (All) and for each individual DT cluster (8, 9, 10, 11, 12 and 13). **b**, Kaplan-Meier estimates of survival before death/censored in the group of EGFR-mutant lung adenocarcinoma patients with significantly upregulated (Z-score > 1.96, $P < 0.05$) DT markers that were identified in the PC9 xenograft treated with osimertinib compared to the patient group, where DT markers showed decreased expression or no significant change ($P > 0.05$). **c**, Sample level enrichment analysis of DT markers, which were identified in the M14 melanoma cells treated with vemurafenib, was performed in BRAF-mutant TCGA melanomas. Expression level was determined for markers of DT clusters (Clusters 5-8 in Fig. 3a and genes in in Supplementary Data 43), for all clusters together (All) and for each individual DT cluster (5,6,7 and 8). **d**, Kaplan-Meier estimates of survival before death/censored in the group of BRAF-mutant melanoma patients (TCGA) with significantly upregulated (Z-score > 1.96, $P < 0.05$) DT markers that were identified in the M14 experiment compared to the group of patients, where DT markers showed decreased expression or no significant change ($P > 0.05$). Survival was calculated based all cluster markers (All) and on the markers of each individual DT cluster (5,6,7 and 8). **e** and **f**, Kaplan-Meier estimates of survival as in (d) performed for GSE65904 and GSE53118 data sets, respectively. Survival was calculated based on the markers of each individual DT cluster (5,6,7 and 8). Patients with upregulated genes (DT markers) survive less in (e), although the *P*-values of survival difference are not significant. The markers were considered to be overexpressed in a patient if the Z-score >1.96 ($P < 0.05$), they are shown in the colors of red; the rest of the patients show the markers either significantly decreased ($P < 0.05$) in colors of blue or not significantly changed (grey). Survival information (in days) is shown for each patient. Univariate Cox regression was used to determine Hazard Ratio (HR) and log rank *P* values.

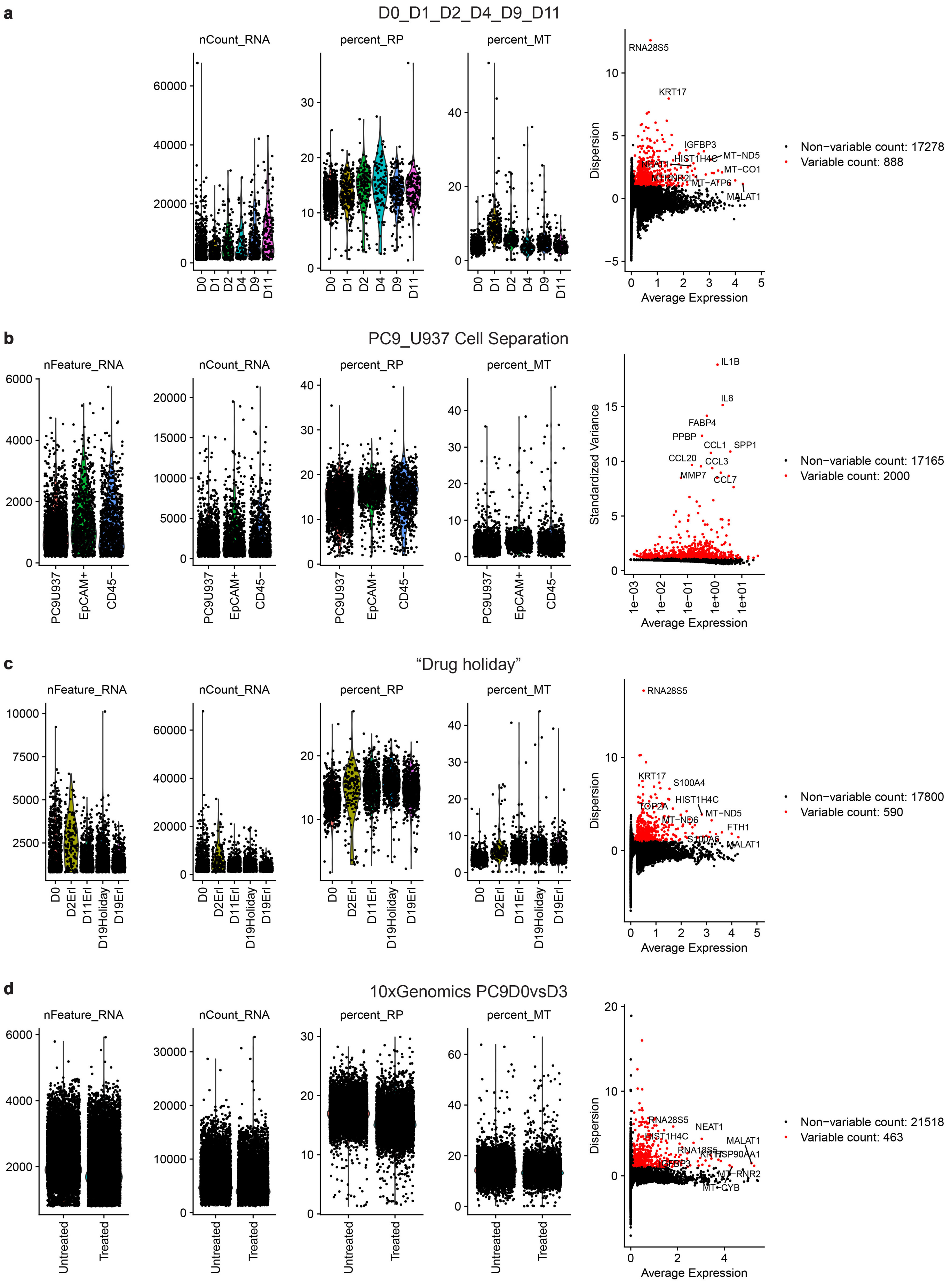
a
D0_D1_D2_D4_D9_D11
nCount_RNA
percent_RP
percent_MT
Dispersion
Average Expression
Non-variable count: 17278
Variable count: 888
b
PC9_U937 Cell Separation
nFeature_RNA
nCount_RNA
percent_RP
percent_MT
Standardized Variance
Average Expression
Non-variable count: 17165
Variable count: 2000
c
"Drug holiday"
nFeature_RNA
nCount_RNA
percent_RP
percent_MT
Dispersion
Average Expression
Non-variable count: 17800
Variable count: 590
d
10xGenomics PC9D0vsD3
nFeature_RNA
nCount_RNA
percent_RP
percent_MT
Dispersion
Average Expression
Non-variable count: 21518
Variable count: 463

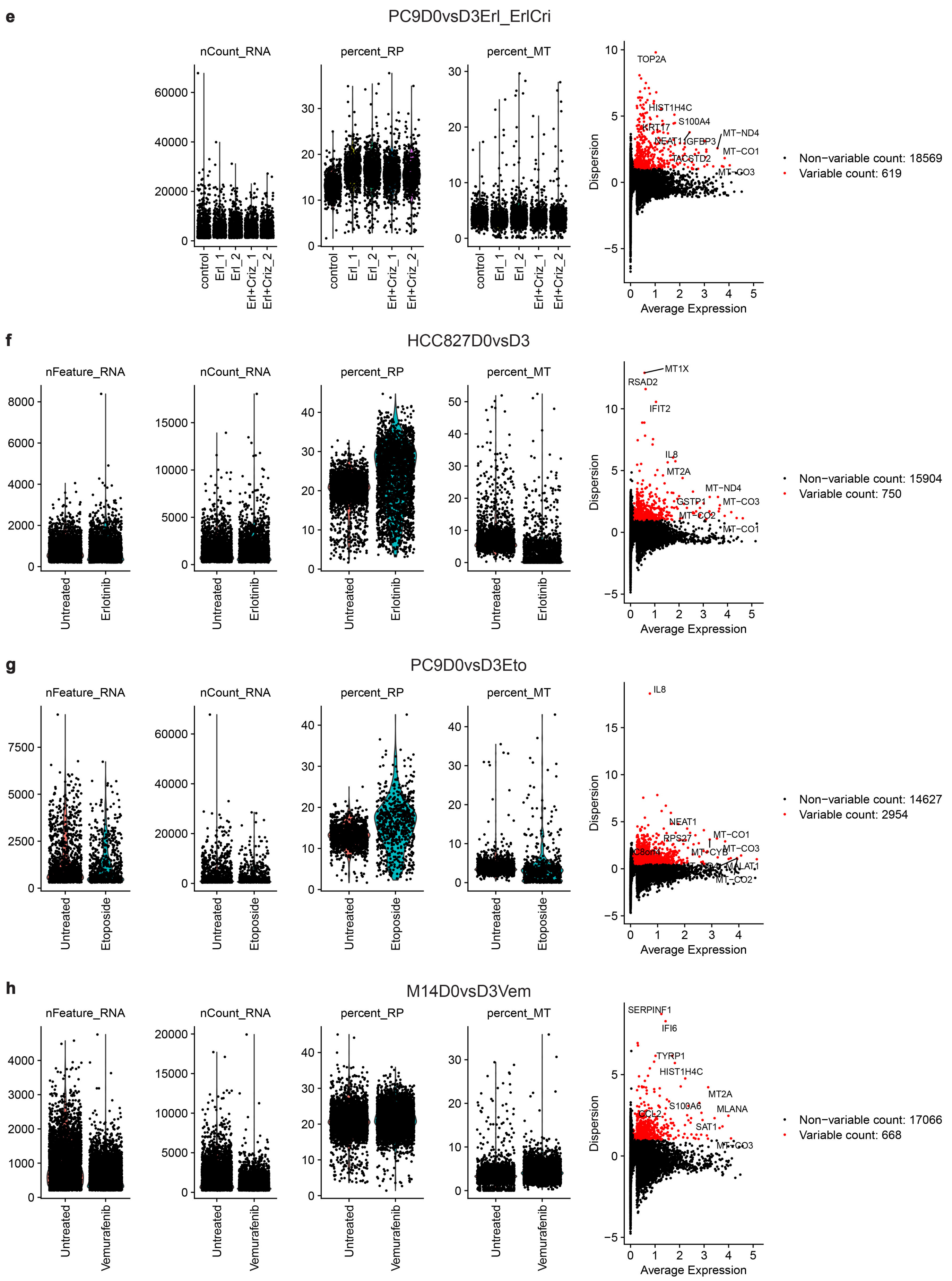
e
PC9D0vsD3Erl_ErlCri
nCount_RNA
percent_RP
percent_MT
control
Erl_1
Erl_2
Erl+Criz_1
Erl+Criz_2
Dispersion
Average Expression
TOP2A
HIST1H4C
S100A4
KRT17
MT−ND4
MT−CO1
TACSTD2
MT−CO3
Non−variable count: 18569
Variable count: 619
f
HCC827D0vsD3
nFeature_RNA
nCount_RNA
percent_RP
percent_MT
Untreated
Erlotinib
MT1X
RSAD2
IFIT2
IL8
MT2A
MT−ND4
GSTP1
MT−CO3
MT−CO2
MT−CO1
Non−variable count: 15904
Variable count: 750
g
PC9D0vsD3Eto
nFeature_RNA
nCount_RNA
percent_RP
percent_MT
Untreated
Etoposide
IL8
NEAT1
RPS27
MT−CO1
MT−CO3
MT−CO2
Non−variable count: 14627
Variable count: 2954
h
M14D0vsD3Vem
nFeature_RNA
nCount_RNA
percent_RP
percent_MT
Untreated
Vemurafenib
SERPINF1
IFI6
TYRP1
HIST1H4C
MT2A
S100A6
MLANA
CCL2
SAT1
MT−CO3
Non−variable count: 17066
Variable count: 668

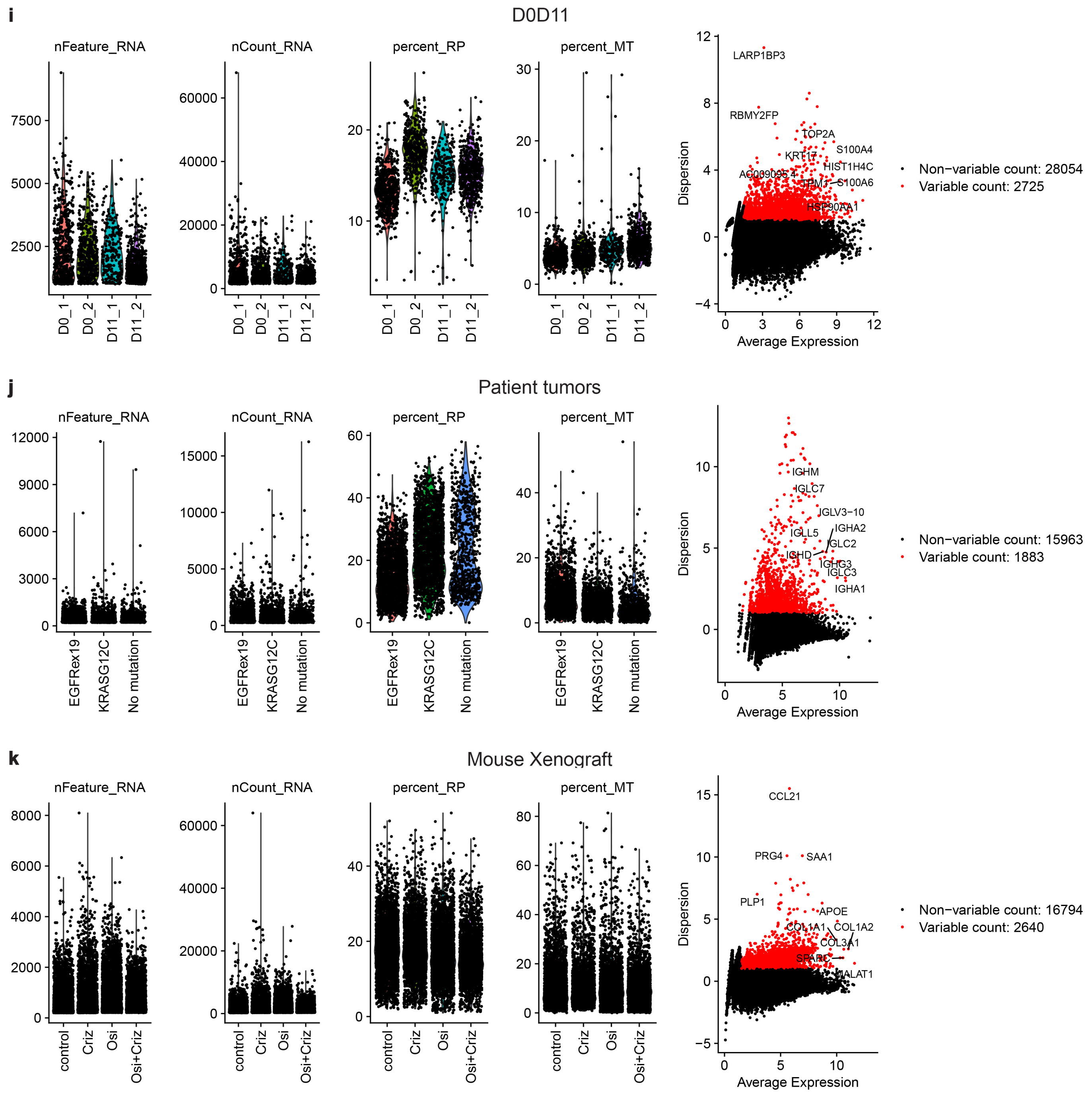

Supplementary Fig. 17. Assessing cell quality in single-cell RNA sequencing experiments. a – k, For each sample set, the quality was assessed using parameters in the Drop-seq core computational pipeline. See also Supplementary Fig. 1c and 12a.

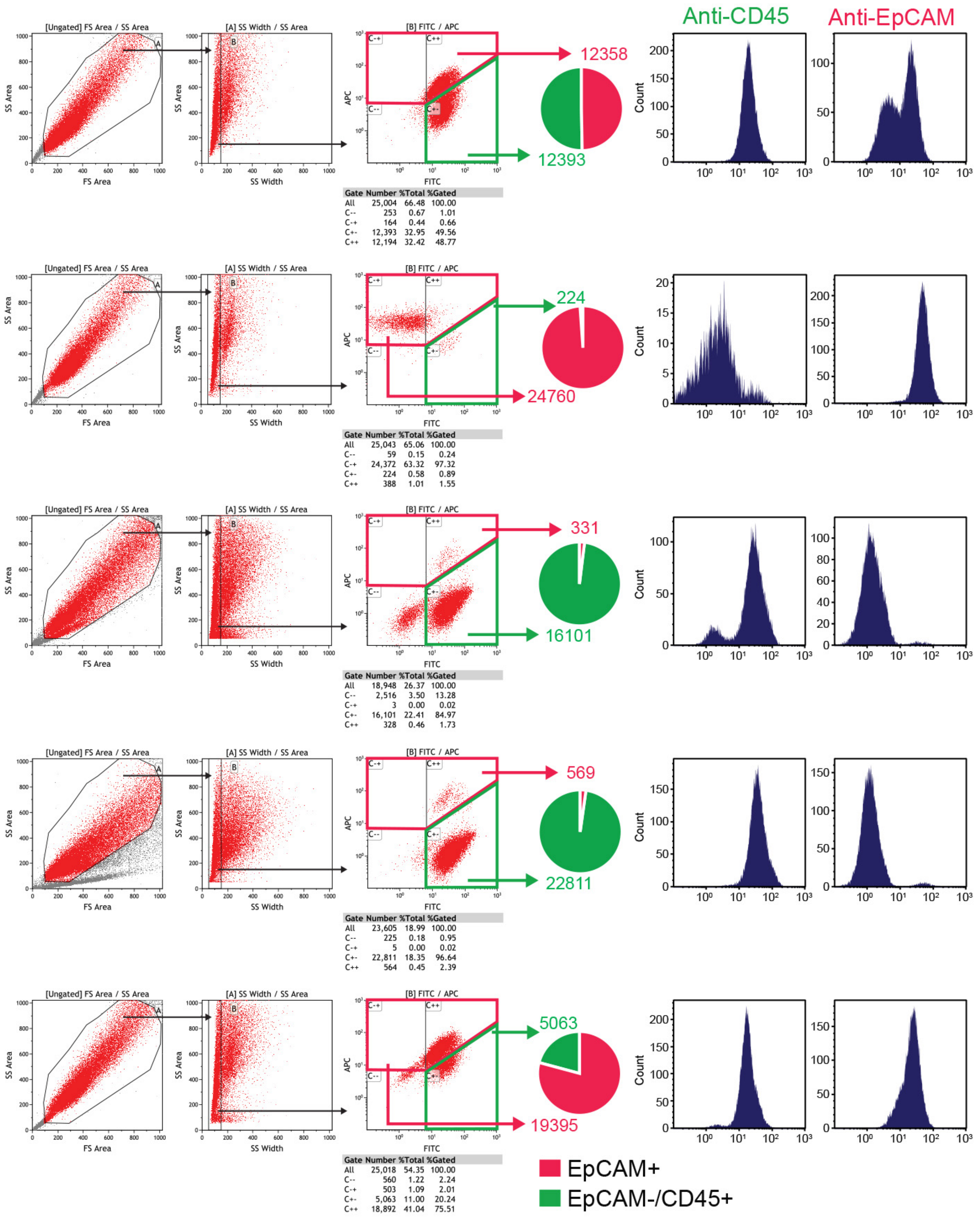

Supplementary Fig. 18. Gating strategies used for flow cytometry. Gating strategy for detection of EpCAM^{+} (red) and EpCAM-negative/CD45-positve (EpCAM^{-}/CD45^{+} green) cells in each sample from the mixtures of PC9 and U937 cells in Supplementary Fig. 3b.

Supplementary Tables

Supplementary Table 1. Number of sequenced cells and genes.

PC9_U937 Cell Separation	# Genes	# Cells
PC9U937	17993	2031
EpCAM+	16256	849
CD45-	16174	897
D0_D1_D2_D4_D9_D11	# Genes	# Cells
D0	16211	756
D1	13047	234
D2	13896	144
D4	12762	99
D9	15575	228
D11	14686	143
"Drug holiday"	# Genes	# Cells
D11Erl	14215	716
D19Holiday	15903	722
D19Erl	13263	716
10xGenomics PC9D0vsD3	# Genes	# Cells
Untreated	20911	12330
Treated	20591	11085
PC9D0vsD3Erl_ErlCri	# Genes	# Cells
control	16211	756
Erl+Criz replicate 1	15742	876
Erl+Criz replicate 2	15505	762
Erl replicate 1	16160	678
Erl replicate 2	16819	945
HCC827D0vsD3	# Genes	# Cells
Untreated	14366	2787
Erlotinib	15989	2591
PC9D0vsD3Eto	# Genes	# Cells
Untreated	16506	1289
Etoposide	14555	684
M14D0vsD3Vem	# Genes	# Cells
Untreated	16540	3462
Vemurafenib	15806	4836
D0D11	# Genes	# Cells
D0_1	16211	756
D0_2	16871	545
D11_1	14206	260
D11_2	14215	716
Patient tumors	# Genes	# Cells
EGFR ex19	14087	2026
KRAS G12C	13758	1694
MULTIPLE	11154	608
Mouse Xenograft	# Genes	# Cells
control	14136	4406
Criz	14316	2977
Osi	15296	3402
Osi+Criz	14420	2953

Supplementary Table 2. Seurat parameters used in scRNA-seq.

Dataset	nFeature_RNA_min	nFeature_RNA_max	FindVariableFeatures	PCAs used	Resolution	Related to
PC9_U937 Cell Separation	>200	<7500	Seurat 2.3.4 (x.low.cutoff = 0.0125, x.high.cutoff = 3, y.cutoff = 0.5, + Default	1:8	0.5	Supplementary Fig. 3 and 4
D0_D1_D2_D4_D9_D11	>800	<7500	nfeatures = 1000	1:10	0.55	Fig. 1
"Drug holiday"	>800	<7500	nfeatures = 2000	1:8	0.55	Fig. 2
10xGenomics PC9D0vsD3	>800	<7500	nfeatures = 2000	1:20	2	Fig. 4
PC9D0vsD3Erl_ErlCri	>800	<7500	nfeatures = 2000	1:12	1.1	Fig. 6
PC9D0vsD3	>800	<7500	nfeatures = 2000	1:10	0.6	Fig. 3
HCC827D0vsD3	>200	<7500	nfeatures = 2000	1:9	0.6	Fig. 3
PC9D0vsD3Eto	>300	<7500	nfeatures = 2000, mean.cutoff = c(0.1, Inf), dispersion.cutoff = c(0.5, Inf)	1:8	0.5	Fig. 3
M14D0vsD3Vem	>200	<7500	nfeatures = 2000	1:7	0.65	Fig. 3
D0D11	>800	<7500	nfeatures = 2000	1:10	0.55	Fig. 1
Patient tumors (whole population)	>200	<5000	SCTransform, vars.to.regress = c("nFeature_RNA", "nCount_RNA")	1:20	0.7	Fig. 8
Patients tumors (Epithelial)	>200	<5000	SCTransform, vars.to.regress = c("nFeature_RNA", "nCount_RNA")	1:25	0.7	Fig. 8
Mouse Xenograft Control,Osi	>300	<4000	nfeatures = 2000	1:10	1.2	Fig. 7a
Mouse Xenograft Control,Criz,Osi,Osi+Criz	>300	<4000	nfeatures = 2000	1:11	1.8	Fig. 7b

nFeature_RNA_min	the minimum number of genes per cell for inclusion this cell in analysis
nFeature_RNA_max	the maximum number of genes per cell for inclusion this cell in analysis
