# Supplementary material for: Single-cell transcriptional changes associated with drug tolerance and response to combination therapies in cancer
Source: Nat Commun. 2021 Mar 12;12:1628. doi: 10.1038/s41467-021-21884-z (PMC7955121; doi:10.1038/s41467-021-21884-z)
Supplement: Supplementary file 3 — Description of Additional Supplementary Files [file 41467_2021_21884_MOESM3_ESM.pdf]

Description of Additional Supplementary Files

File Name: Supplementary Data 1
Description: Markers of resistant clusters in PC9 cells treated with erlotinib for 1, 2, 4, 9, 11 days and untreated. Gene, associated gene name; p_val, p-value; avg_logFC, the natural log of the average fold change between the cluster and the rest of clusters; pct.1, percentage of cells within the cluster expressing the gene; pct.2, percentage of the rest of cells expressing the gene; p_val_adj (or P), non-parametric Wilcoxon rank sum test, adjustments for multiple comparisons were made using Bonferroni correction method. Listed for each cluster are the genes with $P < 0.05$.

File Name: Supplementary Data 2
Description: Markers of resistant clusters in PC9 cells treated with erlotinib for 1, 2, 4, 9 and 11 days or untreated after regressing cell cycle genes. P values, non-parametric Wilcoxon rank sum test, adjustments for multiple comparisons were made using Bonferroni correction method.

File Name: Supplementary Data 3
Description: Top 10 markers of clusters in Velocyto analysis of 10xGenomics data of PC9 cells treated with erlotinib for 3 days and untreated cells. P values, non-parametric Wilcoxon rank sum test, adjustments for multiple comparisons were made using Bonferroni correction method.

File Name: Supplementary Data 4
Description: Genes differentially expressed in PC9 cells treated with erlotinib for 11 days compared to untreated cells, which show significant difference in H3K4me3 enrichment. The genes were identified by Drop-seq. P values are based on Poisson distribution, adjusted for multiple testing using Benjamini-Hochberg FDR method.

File Name: Supplementary Data 5
Description: Top 100 differentially expressed genes in PC9 cells treated with erlotinib for 11 days compared to untreated cells that were identified by Drop-seq. P values, non-parametric Wilcoxon rank sum test, adjustments for multiple comparisons were made using Bonferroni correction method. Genes were first sorted by Adjusted p-value and then ranked according to Log2FoldChange (avg_logFC). pct.1 is untreated cells and pct.2 is Day 11 cells.

File Name: Supplementary Data 6
Description: Top 100 differentially expressed genes in PC9 cells treated with erlotinib for 11 days compared to untreated cells that were identified by bulk RNA-seq. P values are from a likelihood ratio test, which has an asymtotically chi-square test statistic, were adjusted for multiple testing using Benjamini-Hochberg FDR method. Genes were first sorted by Adjusted P values and then ranked according to Log2FoldChange.

File Name: Supplementary Data 7

Description: GSEA of pre-ranked list of Protein/RNA correlation for markers of PC9 resistant states. Normalized enrichment score corresponds to weighted Kolmogorov–Smirnov-like statistic. The FDR was estimated by the Benjamini-Hochberg method.

File Name: Supplementary Data 8
Description: Correlations between protein and gene expression values for markers of PC9 resistant states. Average correlation value across all markers is reported for each cluster at the bottom, as well as an average across all clusters is reported to the right of the cluster groups.

File Name: Supplementary Data 9
Description: Canonical and additional markers of U937 cells identified by Drop-seq. * References for markers previously identified in TPA-treated cells.

File Name: Supplementary Data 10
Description: GSEA of pre-ranked list of Protein RNA correlation for markers of HCC827 resistant cell populations. Normalized enrichment score corresponds to weighted Kolmogorov –Smirnovlike statistic. The FDR was estimated by the Benjamini-Hochberg method.

File Name: Supplementary Data 11
Description: Correlations between protein and gene expression values for markers of HCC827 resistant cell populations. Average correlation value across all markers is reported for each cluster at the bottom, as well as an average across all clusters is reported to the right of the cluster groups.

File Name: Supplementary Data 12
Description: Three EMT modules detected in DT cells. 10xGenomics data of PC9 cells treated with erlotinib for 3 days and untreated cells.

File Name: Supplementary Data 13
Description: Top 30 drugs identified in LINCS analysis as downregulating markers of resistant states in PC9 cells. Normalized enrichment score (NES) corresponds to weighted Kolmogorov– Smirnov-like statistic, FDR was computed by comparing the tails of the observed and null distributions. Data was sorted by Normalized enrichment scores and then by FDR-adjusted P values. PC9 cells were treated with erlotinib for 1, 2, 4, 9 or 11 days. Cluster 6 did not identify any drug and is not shown.

File Name: Supplementary Data 14
Description: Top 30 drugs identified in LINCS analysis as downregulating genes that are the top upregulated in PC9 cells treated with erlotinib for 11 days compared to untreated as identified by bulk RNA-seq. Normalized enrichment score (NES) corresponds to weighted Kolmogorov–Smirnov-like statistic, FDR was computed by comparing the tails of the observed and null distributions. Data was sorted by Normalized enrichment scores and then by FDR-adjusted P values.

File Name: Supplementary Data 15

Description: Top 30 drugs identified in LINCS analysis as downregulating markers of PC9 cell populations tolerant to erlotinib. Normalized enrichment score (NES) corresponds to weighted Kolmogorov–Smirnov-like statistic, FDR was computed by comparing the tails of the observed and null distributions. Data was sorted by Normalized enrichment scores and then by FDR-adjusted P values. PC9 cells were treated with erlotinib for 3 days. Markers were identified by Drop-seq.

File Name: Supplementary Data 16
Description: Top 30 drugs identified in LINCS analysis as downregulating markers of HCC827 cell populations tolerant to erlotinib. Normalized enrichment score (NES) corresponds to weighted Kolmogorov–Smirnov-like statistic, FDR was computed by comparing the tails of the observed and null distributions. Data was sorted by Normalized enrichment scores and then by FDR-adjusted P values. HCC827 cells were treated with erlotinib for 3 days

File Name: Supplementary Data 17
Description: Drugs upregulating markers of DT states identified in PC9 cells and mapped on biological pathways. Normalized enrichment score (NES) corresponds to weighted Kolmogorov–Smirnov-like statistic, FDR was computed by comparing the tails of the observed and null distributions. LINCS GSEA data is presented for the top drug in each list, with the total number of significant drugs (Adjusted p-value < 0.05) indicated in bold in parentheses.

File Name: Supplementary Data 18
Description: Top 30 drugs identified in LINCS analysis as downregulating markers of PC9 cell populations tolerant to etoposide. Normalized enrichment score (NES) corresponds to weighted Kolmogorov–Smirnov-like statistic, FDR was computed by comparing the tails of the observed and null distributions. Data was sorted by Normalized enrichment scores and then by FDR-adjusted P values. PC9 cells were treated with etoposide for 3 days.

File Name: Supplementary Data 19
Description: Top 30 drugs identified in LINCS analysis as downregulating markers of M14 cell populations tolerant to vemurafenib. Normalized enrichment score (NES) corresponds to weighted Kolmogorov–Smirnov-like statistic, FDR was computed by comparing the tails of the observed and null distributions. Data was sorted by Normalized enrichment scores and then by FDR-adjusted P values. M14 cells were treated with vemurafenib for 3 days.

File Name: Supplementary Data 20
Description: Markers characterizing each cluster arising from the combination of erlotinib and crizotinib or erlotinib alone. P values are from Seurat, which uses a non-parametric Wilcoxon rank sum test, adjustments for multiple comparisons were made using Bonferroni correction method.

File Name: Supplementary Data 21

Description: Top 20 drug candidates targeting Erl+Criz-tolerant clusters and Erl+Criz-sensitive clusters using LINCS GSEA. Data was sorted by Normalized enrichment scores and then by FDR-adjusted P values.

File Name: Supplementary Data 22
Description: Top 30 drugs identified in LINCS analysis as downregulating markers of PC9 xenograft cell populations tolerant to osimertinib. Normalized enrichment score (NES) corresponds to weighted Kolmogorov –Smirnov-like statistic, FDR was computed by comparing the tails of the observed and null distributions. Data was sorted by Normalized enrichment scores and then by FDR-adjusted P values.

File Name: Supplementary Data 23
Description: Patient characteristics.

File Name: Supplementary Data 24
Description: Markers used for cell type annotation.

File Name: Supplementary Data 25
Description: Markers of cell populations (clusters) identified through analysis of donor and NSCLC samples. P values are from Seurat, which uses a non-parametric Wilcoxon rank sum test, adjustments for multiple comparisons were made using Bonferroni correction method.

File Name: Supplementary Data 26
Description: Markers of the epithelial subset of cell populations (clusters) identified through analysis of donor and NSCLC samples. P values are from Seurat, which uses a non-parametric Wilcoxon rank sum test, adjustments for multiple comparisons were made using Bonferroni correction method.

File Name: Supplementary Data 27
Description: Markers of the cancer cell cluster from patient with EGFRex19 mutation (Cluster 4) versus donor AT2 cell clusters. Markers discussed in the main text are in bold. A, Markers of the EGFRex19 mutant Cluster 4. B, Markers of donor AT2 cell Clusters. P values are from Seurat, which uses a non-parametric Wilcoxon rank sum test, adjustments for multiple comparisons were made using Bonferroni correction method.

File Name: Supplementary Data 28
Description: Top drugs identified in LINCS analysis as downregulating markers of EGFRex19 and KRASG12C patient tumors. Normalized enrichment score corresponds to weighted Kolmogorov–Smirnov-like statistic, FDR was computed by comparing the tails of the observed and null distributions.

File Name: Supplementary Data 29

Description: Oligonucleotide sequences.

File Name: Supplementary Data 30
Description: Average expression values for each gene per cluster of PC9 cells treated with erlotinib for 1, 2, 4, 9, 11 days and untreated.

File Name: Supplementary Data 31
Description: Differentially expressed genes (cluster markers) of PC9 cells treated with erlotinib for 1, 2, 4, 9, 11 days and untreated. P values are from Seurat, which uses a non-parametric Wilcoxon rank sum test, adjustments for multiple comparisons were made using Bonferroni correction method.

File Name: Supplementary Data 32
Description: Average expression values for each gene per cluster of PC9 cells in “drug holiday” experiment with erlotinib.

File Name: Supplementary Data 33
Description: Differentially expressed genes (cluster markers) of PC9 cells in “drug holiday” experiment with erlotinib. P values are from Seurat, which uses a non-parametric Wilcoxon rank sum test, adjustments for multiple comparisons were made using Bonferroni correction method

File Name: Supplementary Data 34
Description: Average expression values for each gene per cluster of PC9 cells treated with erlotinib for 3 days or left untreated using Drop-seq data.

File Name: Supplementary Data 35
Description: Differentially expressed genes (cluster markers) of PC9 cells treated with erlotinib for 3 days or left untreated using Dropseq data. P values are from Seurat, which uses a non-parametric Wilcoxon rank sum test, adjustments for multiple comparisons were made using Bonferroni correction method.

File Name: Supplementary Data 36
Description: Average expression values for each gene per cluster of PC9 cells treated with erlotinib for 3 days or left untreated using 10xGenomics data.

File Name: Supplementary Data 37
Description: Differentially expressed genes (cluster markers) of PC9 cells treated with erlotinib for 3 days or left untreated using 10xGenomics data. P values are from Seurat, which uses a nonparametric Wilcoxon rank sum test, adjustments for multiple comparisons were made using Bonferroni correction method.

File Name: Supplementary Data 38
Description: Average expression values for each gene per cluster of PC9 cells treated for 3 days with erlotinib, alone and in combination with crizotinib, or left untreated.

File Name: Supplementary Data 39
Description: Differentially expressed genes (cluster markers) of PC9 cells treated for 3 days with erlotinib, alone and in combination with crizotinib, or left untreated. P values are from Seurat, which uses a non-parametric Wilcoxon rank sum test, adjustments for multiple comparisons were made using Bonferroni correction method.

File Name: Supplementary Data 40
Description: Average expression values for each gene per cluster of HCC827 cells treated with erlotinib for 3 days or left untreated.

File Name: Supplementary Data 41
Description: Differentially expressed genes (cluster markers) of HCC827 cells treated with erlotinib for 3 days or left untreated. P values are from Seurat, which uses a non-parametric Wilcoxon rank sum test, adjustments for multiple comparisons were made using Bonferroni correction method.

File Name: Supplementary Data 42
Description: Average expression values for each gene per cluster of M14 cells treated with vemurafenib for 3 days or left untreated.

File Name: Supplementary Data 43
Description: Differentially expressed genes (cluster markers) of M14 cells treated with vemurafenib for 3 days or left untreated. P values are from Seurat, which uses a non-parametric Wilcoxon rank sum test, adjustments for multiple comparisons were made using Bonferroni correction method.

File Name: Supplementary Data 44
Description: Average expression values for each gene per cluster of PC9 cells treated with etoposide for 3 days or left untreated.

File Name: Supplementary Data 45
Description: Differentially expressed genes (cluster markers) of PC9 cells treated with etoposide for 3 days or left untreated. P values are from Seurat, which uses a non-parametric Wilcoxon rank sum test, adjustments for multiple comparisons were made using Bonferroni correction method.

File Name: Supplementary Data 46
Description: Average expression values for each gene per cluster of the original PC9:U937 sample (PC9 cells mixed with differentiated U937 cells), and EpCAM-positive and CD45-negative cell populations, which were generated from the PC9:U937 sample.

File Name: Supplementary Data 47
Description: Differentially expressed genes (cluster markers) of the original PC9:U937 sample (PC9 cells mixed with differentiated U937 cells), and EpCAM-positive and CD45-negative cell populations, which were generated from the PC9:U937 sample. P values are from Seurat, which uses a non-parametric Wilcoxon rank sum test, adjustments for multiple comparisons were made using Bonferroni correction method.

File Name: Supplementary Data 48
Description: Average expression values for each gene per cluster of PC9 mouse xenografts treated with osimertinib or vehicle.

File Name: Supplementary Data 49
Description: Differentially expressed genes (cluster markers) of PC9 mouse xenografts treated with osimertinib or vehicle. P values are from Seurat, which uses a non-parametric Wilcoxon rank sum test, adjustments for multiple comparisons were made using Bonferroni correction method.

File Name: Supplementary Data 50
Description: Average expression values for each gene per cluster of PC9 mouse xenografts treated with osimertinib, crizotinib, their combination or vehicle.

File Name: Supplementary Data 51
Description: Differentially expressed genes (cluster markers) of PC9 mouse xenografts treated with osimertinib, crizotinib, their combination or vehicle.

File Name: Supplementary Data 52
Description: Average expression values for each gene per cluster identified through analysis of donor and NSCLC samples.

File Name: Supplementary Data 53
Description: Differentially expressed genes (cluster markers) identified through analysis of donor and NSCLC samples. P values are from Seurat, which uses a non-parametric Wilcoxon rank sum test, adjustments for multiple comparisons were made using Bonferroni correction method.

File Name: Supplementary Data 54
Description: Average expression values for each gene per cluster of the epithelial subset identified through analysis of donor and NSCLC samples.

File Name: Supplementary Data 55

Description: Differentially expressed genes (cluster markers) of the epithelial subset identified through analysis of donor and NSCLC samples. P values are from Seurat, which uses a non-parametric Wilcoxon rank sum test, adjustments for multiple comparisons were made using Bonferroni correction method
