# Supplementary material for: Single-cell transcriptional changes associated with drug tolerance and response to combination therapies in cancer
Source: Nat Commun. 2021 Mar 12;12:1628. doi: 10.1038/s41467-021-21884-z (PMC7955121; doi:10.1038/s41467-021-21884-z)
Supplement: Supplementary file 6 — Reporting Summary [file 41467_2021_21884_MOESM6_ESM.pdf]

nature research

Corresponding author(s): Elizaveta V Benevolenskaya

Last updated by author(s): Dec 24, 2020

# Reporting Summary

Nature Research wishes to improve the reproducibility of the work that we publish. This form provides structure for consistency and transparency in reporting. For further information on Nature Research policies, see our Editorial Policies and the Editorial Policy Checklist.

## Statistics

For all statistical analyses, confirm that the following items are present in the figure legend, table legend, main text, or Methods section.

| n/a | Confirmed | |
|---|---|---|
| ☐ | ☒ | The exact sample size ($n$) for each experimental group/condition, given as a discrete number and unit of measurement |
| ☐ | ☒ | A statement on whether measurements were taken from distinct samples or whether the same sample was measured repeatedly |
| ☐ | ☒ | The statistical test(s) used AND whether they are one- or two-sided<br>*Only common tests should be described solely by name; describe more complex techniques in the Methods section.* |
| ☐ | ☒ | A description of all covariates tested |
| ☐ | ☒ | A description of any assumptions or corrections, such as tests of normality and adjustment for multiple comparisons |
| ☐ | ☒ | A full description of the statistical parameters including central tendency (e.g. means) or other basic estimates (e.g. regression coefficient) AND variation (e.g. standard deviation) or associated estimates of uncertainty (e.g. confidence intervals) |
| ☐ | ☒ | For null hypothesis testing, the test statistic (e.g. $F$, $t$, $r$) with confidence intervals, effect sizes, degrees of freedom and $P$ value noted<br>*Give P values as exact values whenever suitable.* |
| ☒ | ☐ | For Bayesian analysis, information on the choice of priors and Markov chain Monte Carlo settings |
| ☒ | ☐ | For hierarchical and complex designs, identification of the appropriate level for tests and full reporting of outcomes |
| ☐ | ☒ | Estimates of effect sizes (e.g. Cohen's $d$, Pearson's $r$), indicating how they were calculated |

*Our web collection on statistics for biologists contains articles on many of the points above.*

## Software and code

Policy information about availability of computer code

| | |
|---|---|
| Data collection | The Drop-seq pipeline R version 3.5.0 and Seurat version 2.3.4 or R version 3.5.3 and Seurat version 3.1.2 (https://satijalab.org/seurat) and 10x Genomics pipeline used version 3.5.3 and Seurat version 3.1.2. |
| Data analysis | Monocle3 (https://githu b.com/cole-trapnell-lab/monocle3), Scanpy function for PAGA (https://scanpy.readthedocs.io/en/stable/), DESeq2 (https://bioconductor.org/packages/release/bioc/html/DESeq2.html), SICER program (http://home.gwu.edu/~wpeng/Software.htm), Azure Capture cSeries software (https://www.azurebiosystems.com/software-downloads/azurespot/), STORM algorithm (http://rulai.cshl.edu/storm/storm-examples.shtml), RNA FISH Probe Designer (www.biosearchtech.com/stellarisdesigner), GPP sgRNA Designer (https://portals.broadinstitute.org/gpp/public/analysis-tools/sgrna-design), Kaluza Analysis software (https://www.beckman.com/flow-cytometry/software/kaluza/downloads), ZEN 2.6 pro software (https://www.micro-shop.zeiss.com/en/us/system/zen+software/software+zen/zen+-+basic+software/410135-1002-260), Fiji Analyze Particles program (https://imagej.net/Particle_Analysis). |

For manuscripts utilizing custom algorithms or software that are central to the research but not yet described in published literature, software must be made available to editors and reviewers. We strongly encourage code deposition in a community repository (e.g. GitHub). See the Nature Research guidelines for submitting code & software for further information.

## Data

Policy information about availability of data

All manuscripts must include a data availability statement. This statement should provide the following information, where applicable:

- Accession codes, unique identifiers, or web links for publicly available datasets
- A list of figures that have associated raw data
- A description of any restrictions on data availability

Both the raw and processed scRNA-seq data has been deposited to the database Gene Expression Omnibus (GEO) (https://www.ncbi.nlm.nih.gov/geo) in

SuperSeries under the accession number GSE149383. RNA-seq data is available under GEO GSE148465. ChIP-seq data is available under GEO GSE148461. Previously published data included: bulk RNA from the lung GSE31210 and from melanoma TCGA SKCM, GSE65904 and GSE53118; the donor lung tissue scRNA-seq data from GSE130148 (GSM3732848 for sample 1, GSM3732850 for sample 2 and GSM3732854 for sample 3); and LINCS database data. The whole-genome data on proteomics to RNA-seq correlation was obtained from the CCLE study (Nusinow et al., 2020). Source data is available as a Source Data file. The authors declare that all data supporting the findings of this study are available within the Article and its Supplementary Information, Supplementary files or from the corresponding author upon reasonable request. This data supports Fig. 1-8.

# Field-specific reporting

Please select the one below that is the best fit for your research. If you are not sure, read the appropriate sections before making your selection.

- [x] Life sciences
- [ ] Behavioural & social sciences
- [ ] Ecological, evolutionary & environmental sciences

For a reference copy of the document with all sections, see nature.com/documents/nr-reporting-summary-flat.pdf

# Life sciences study design

All studies must disclose on these points even when the disclosure is negative.

| | |
|---|---|
| Sample size | We generated 34 scRNA-seq samples, where we sequenced 51,421 cancer cell line cells, 13,738 xenograft tumor cells and 4328 patient tumor cells. The highest number of cells was 4,836 and the lowest number was 99. Seurat was used to run statistical tests on each sample. For each gene, fdr-corrected P values are reported in Supplementary Data. |
| Data exclusions | In scRNA-seq, we empirically determined the cutoff for the minimum number of genes per cell (nFeature_RNA), which was not high enough to disproportionately filter out the cells but also not low enough to affect the cell distribution by inclusion of low-quality cells, while avoiding rare cell doublets by setting the nFeature_RNA_max. These criteria were not pre- established as the quality of cells varied between different biological models and treatments. The utilized cuttoffs are reported in Supplementary Table 2. For all samples, mitochondrial genes were removed after the mapping. For patient samples, preudogenes were removed along with the mitochondrial genes. We removed genes expressed in less than 3 single cells. |
| Replication | The critical experiments such as survival assays in erlotinib-treated PC9 cells, SERPINE1 and TACSTD2 shRNA knockdowns in PC9, immunostaining for CYP1B1, SERPINE1 and TACSTD2, RNA extraction and RT-qPCR analysis were successfully reproduced by at least two investigators. Cell survival assays were successfully replicated in most cases at least 3 times, and in a few cases 2 times. The 37-day long colony formation assay in M14 cell line was an exception as it was performed as three biological replicates run in parallel once. scRNA-seq were successfully replicated for PC9 D0 and D11 treatment with erlotinib, and for D3 treatment with erlotinib and crizotinib. In addition, PC9 D0 and D11 bulk RNA-seq data and D3 10x Genomics data were integrated to validate findings. |
| Randomization | Wells with seeded cells were selected randomly to be treated with compounds. Bulk samples were collected from multiple plates, thus averaging the batch effect. Mouse was assigned to a random treatment group once the tumor reached 200-240 mm3. |
| Blinding | The investigators were not blinded to sample annotation because the experiments were designed by the same person who performed them. |

# Reporting for specific materials, systems and methods

We require information from authors about some types of materials, experimental systems and methods used in many studies. Here, indicate whether each material, system or method listed is relevant to your study. If you are not sure if a list item applies to your research, read the appropriate section before selecting a response.

## Materials & experimental systems

| n/a | Involved in the study | |
|---|---|---|
| ☐ | ☒ | Antibodies |
| ☐ | ☒ | Eukaryotic cell lines |
| ☒ | ☐ | Palaeontology and archaeology |
| ☐ | ☒ | Animals and other organisms |
| ☒ | ☐ | Human research participants |
| ☒ | ☐ | Clinical data |
| ☒ | ☐ | Dual use research of concern |

## Methods

| n/a | Involved in the study | |
|---|---|---|
| ☐ | ☒ | ChIP-seq |
| ☐ | ☒ | Flow cytometry |
| ☒ | ☐ | MRI-based neuroimaging |

## Antibodies

| | |
|---|---|
| Antibodies used | Cell separation was performed with anti-EpCAM magnetic microbeads (human CD326, Miltenyi Biotec Cat. 130-061-101), or anti-CD45 magnetic microbeads (Miltenyi Biotec Cat. 130-045-801). Flow cytometry was performed with anti-APC-EpCAM (Miltenyi Biotec, Cat. 130-111-000) or anti-FITC-CD45 (Miltenyi Biotec, Cat. 130-110-631) antibodies. Rabbit polyclonal antibodies were used for CYP1B1 (Thermo Fisher Scientific, Cat. PIPA528040, 1 mg/ml) at a 1:500 dilution. Mouse antibodies were used for TACSTD2 (DSHB, Cat. CPTC-TACSTD2-1-s, 36 µg/ml) at a 1:18 dilution and for SERPINE1 (BD, clone 41, BDB612024, 250 µg/mL) at a 1:50 dilution. Alexa Fluor 488 goat anti-rabbit and anti-mouse IgG (H+L) antibodies (Thermo Fisher Scientific) were diluted 1:200. Mouse |

| | |
|---|---|
| | IgG1 antibodies were used for SERPINE1 (R&D Systems Inc ., Cat. MAB1786-SP, 0.1 µg/mL) and vinculin (Sigma, Cat. V9131, at 1:15,000). Rabbit anti-H3K4me3 (EMD Millipore, Cat. 07-473) antibodies were used for ChIP-seq. |
| Validation | The antibodies were validated by scRNA-seq of sorted populations. We validated the TACSTD2, SERPINE1 and CYP1B1 by shRNAs specific to these genes. The vinculin antibody has been described in 1077 papers. H3K4me3 antibody was validated by knockdown and overexpression of KDM5A followed by WB or immunostaining. Anti-EpCAM, Miltenyi Biotec website: data are available from references (Moldenhauer, G. et al. (1987) Epithelium specific surface glycoprotein of Mr 34,000 is a widely distributed human carcinoma marker. Br. J. Cancer 56: 714-721; Sheridan, C. et al. (2006) CD44+/CD24- breast cancer cells exhibit enhanced invasive properties: an early step necessary for metastasis. Breast Cancer Res. 8(5): R59; Metsuyanim, S. et al. (2009) Expression of stem cell markers in the human fetal kidney. PLoS One 4(68): e6709). Anti-CD45, Miltenyi Biotec website: the antibody was compared to commercially available hybridoma clones in flow cytometry analysis; in order to compare the epitope specificity of an antibody, the clone being used is compared with other known clones recognizing the same antigen in a competition assay, cells were incubated with an excess of purified unconjugated CD45 antibody followed by staining with fluorochrome-conjugated antibodies of other known clones against the same marker, and based on the fluorescence signal obtained, the clones were identified as recognizing completely overlapping (++), partially overlapping (+), or completely different epitopes (-) of the marker (Vykoukal, J. et al. (2008) Enrichment of putative stem cells from adipose tissue using dielectrophoretic field-flow fractionation. Lab Chip 8(8): 1386-1393; and Kurian, L. et al. (2013) Conversion of human fibroblasts to angioblast-like progenitor cells. Nat. Methods 10(1): 77-83). TACSTD2, from DSHB and NICHD websites: CPTC-TACSTD2-1 antibody pairing activity was evaluated by using purifed cognate antigens by Surface Plasmon Resonance (ProteOn XPR36) and Indirect ELISA (ie, binding of Antibody to Antigen coated plate). SERPINE1, BD website: QC testing was performed in human; Western blot analysis was reported on human endothelial cell lysate, with the antibody dilution from 1:2500 to 1:10000. CYP1B1, Invitrogen website: the antibody is for human and mouse species; immunofluorescent analysis was performed in methanol-fixed HeLa cells using this antibody at a 1:200 dilution. SERPINE1, R&D Systems Inc.: the antibody detects human Serpin E1 in direct ELISAs and Western blots; in direct ELISAs, no cross-reactivity with recombinant human Serpin A1, A3, A4, A8, C1, F1, F2, I1, I2, recombinant mouse Serpin D1 or E2 is observed; neutralization was measured by its ability to neutralize Recombinant Human Serpin E1/PAI-1 (0.25 µg/mL, Catalog # 1786-PI) inhibition of Recombinant Human u Plasminogen Activator (uPA)/Urokinase (0.1 µg/mL, Catalog # 1310-SE) cleavage of the fluorogenic peptide substrate Z-GGR-AMC (100 µM), where the Neutralization Dose (ND50) is typically 0.3 µg/mL (References: 1. Silverman, G.A. et al. (2001) J. Biol. Chem. 276:33293. 2. Stefansson, S. et al. (2003) Curr. Pharm. Des. 9:1545. 3. Duffy, M.J. (2002) Clin. Chem. 48:1194. 4. Juhan-Vague, I. et al. (2003) J. Thromb. Haemost. 1:1575. 5. Harbeck, N. et al. (2002) Clin. Breast Cancer 3:196. 6. Pannekoek, H. et al. (1986) EMBO J. 5:2539. 7. Ginsburg, D. et al. (1986) J. Clin. Invest. 78:1673. 8. Wang, Z. et al. (1996) Biochemistry 35:16443. 9. Stromqvist, M. et al. (1994) Protein Expr. Purif. 5:309). Vinculin, Sigma website: monoclonal anti-vinculin specifically stains vinculin at cell-cell and cell-substrate contacts in tissue and cultured cells using indirect immunofluorescent labeling; the antibody reacts with vinculin of many species, good reactivity is obtained with human, bovine, chicken, dog, rat, mouse, turkey, and Xenopus; the antibody shows cross reactivity with smooth muscle metavinculin; specifically labels vinculin at cell-cell and cell-substrate contacts (References: Somatic inactivation of the PHD2 prolyl hydroxylase causes polycythemia and congestive heart failure Minamishima YA, et al. Blood 111(6), 3236-3244, (2008); Prognostic significance of epidermal growth factor receptor phosphorylation and mutation in head and neck squamous cell carcinoma Hama T, et al. Oncologist 14(9), 900-908, (2009)) |

# Eukaryotic cell lines

Policy information about cell lines

| | |
|---|---|
| Cell line source(s) | PC9 (Sigma), HCC827 (ATCC), H1975 (ATCC), M14 (Division of Cancer Treatment and Diagnosis (DCTD) Tumor Repository), U937 (W. Kaelin, DFCI). U937 cell line is available from ATCC. |
| Authentication | The U937 leukemia cell line was authenticated by short tandem repeat analysis at the UIC Genomics Core cell line authentication service. |
| Mycoplasma contamination | All cell lines have been tested negative for mycoplasma contamination. Other cell lines were directly received from corresponding vendor and were not authenticated by us since they were used within several passages and follwoing best laboratory practices. |
| Commonly misidentified lines (See ICLAC register) | Not used. |

# Animals and other organisms

Policy information about studies involving animals; ARRIVE guidelines recommended for reporting animal research

| | |
|---|---|
| Laboratory animals | Female athymic nude (nu/nu) 6-weeks-old mice (Charles River Laboratories) were housed at constant temperature and humidity with a 12-hr light cycle. |
| Wild animals | No wild animals were involved. |
| Field-collected samples | No samples were collected from the field. |
| Ethics oversight | The mouse experiments described in this study were approved by the University of Illinois at Chicago Office of Animal Care and Institutional Biosafety Committee (OACIB) (institutional Animal Welfare Assurance No. A3460.01). |

Note that full information on the approval of the study protocol must also be provided in the manuscript.

# ChIP-seq

## Data deposition

☒ Confirm that both raw and final processed data have been deposited in a public database such as GEO.

☒ Confirm that you have deposited or provided access to graph files (e.g. BED files) for the called peaks.

| | |
|---|---|
| Data access links<br>*May remain private before publication.* | GEO ID GSE148461 ( https://www.ncbi.nlm.nih.gov/geo/query/acc.cgi?acc=GSE148461) |
| Files in database submission | GSE148461_H3K4me3_D00.island.txt.gz<br>GSE148461_H3K4me3_D11.island.txt.gz |
| Genome browser session<br>(e.g. UCSC) | *Provide a link to an anonymized genome browser session for "Initial submission" and "Revised version" documents only, to enable peer review. Write "no longer applicable" for "Final submission" documents.* |

## Methodology

| | |
|---|---|
| Replicates | Two replicates were prepared for each cell treatment. |
| Sequencing depth | Single-end read sequencing was performed on HiSeq 2500. |
| Antibodies | ChIP was performed with anti-H3K4me3 (EMD Millipore, Cat. 07-473) antibodies. |
| Peak calling parameters | After FastQC quality filtering, short reads were uniquely aligned allowing at best two mismatches to the reference genome GRCh37.p13 (Ensembl release 74) using the BOWTIE program (v2.3.0). Peak detection was performed with the Spatial Clustering for Identification of ChIP-Enriched Regions (SICER) algorithm. Reference DNA was the total genomic DNA sample. |
| Data quality | Total of 33,688 peaks (FDR 5%, 5-fold enrichment) of H3K4me3 were detected in D0 samples, and 36,456 H3K4me3peaks were detected in D11 samples. |
| Software | Differential enrichment of H3K4me3 in Day 11 versus untreated cells was determined using SICER program script “SICER-df.sh”. |

# Flow Cytometry

## Plots

Confirm that:

☒ The axis labels state the marker and fluorochrome used (e.g. CD4-FITC).

☒ The axis scales are clearly visible. Include numbers along axes only for bottom left plot of group (a 'group' is an analysis of identical markers).

☒ All plots are contour plots with outliers or pseudocolor plots.

☒ A numerical value for number of cells or percentage (with statistics) is provided.

## Methodology

| | |
|---|---|
| Sample preparation | U937 cells were placed in differentiation medium, RPMI, 10% FBS, PS, containing 50 nM 12-O-tetradecanoylphorbol-13-acetate (TPA) for 72 hrs, trypsinized and mixed with trypsinized PC9 cells at 1:1 ratio. The PC9 cells were grown in regular growth medium. Media was changed 48 hrs before cell collection. The mixed cells were split into three aliquots. Two aliquots were used for purification of PC9 cells by either positive selection with anti-EpCAM magnetic microbeads, or negative selection with anti-CD45 magnetic microbeads. The magnetic separation was performed using MS MACS columns and MACS separator following manufacture protocols (Miltenyi Biotec). An aliquot of each cell suspension was fixed in 70% ethanol and analyzed by flow cytometry using anti-APC-EpCAM or anti-FITC-CD45 antibodies. |
| Instrument | Data acquisition was performed in a Gallios Flow Cytometer (Beckman Coulter). |
| Software | Data was analyzed using the Kaluza Analysis Software (Beckman Coulter) |
| Cell population abundance | All stainings were performed per 300.000 cells. Purity was determined by scRNA-seq and known markers of monocytes and marcophages (transcripts). |
| Gating strategy | Each sample was split in aliquots that were used for either positive selection with anti-EpCAM magnetic microbeads, or negative selection with anti-CD45 magnetic microbeads. |

☒ Tick this box to confirm that a figure exemplifying the gating strategy is provided in the Supplementary Information.
