# Supplementary material for: Single-cell transcriptional changes associated with drug tolerance and response to combination therapies in cancer
Source: Nat Commun. 2021 Mar 12;12:1628. doi: 10.1038/s41467-021-21884-z (PMC7955121; doi:10.1038/s41467-021-21884-z)
Supplement: Supplementary file 7 — Source Data [file 41467_2021_21884_MOESM7_ESM.pdf]

Single-cell transcriptional changes associated with drug tolerance and response to combination therapies in cancer

Alexandre F. Aissa[1], Abul B.M.M.K. Islam[1, 2], Majd M. Ariss[1], Cammille C. Go[1], Alexandra E. Rader[1], Ryan D. Conrardy[1], Alexa M. Gajda[1], Carlota Rubio-Perez[3], Klara Valyi-Nagy[4], Mary Pasquinelli[5], Lawrence E. Feldman[5], Stefan J. Green[6], Nuria Lopez-Bigas[3], Maxim V. Frolov[1] and Elizaveta V. Benevolenskaya[1*].

[1] Department of Biochemistry and Molecular Genetics, University of Illinois at Chicago 900 S. Ashland Ave, Chicago 60607, USA
[2] Department of Genetic Engineering and Biotechnology, University of Dhaka, Dhaka 1000, Bangladesh
[3] Biomedical Genomics Lab, Institute for Research in Biomedicine (IRB), Barcelona 08003, Spain
[4] Department of Pathology, University of Illinois at Chicago, 840 South Wood Street, Chicago, IL, 60612, USA
[5] Department of Medicine, Section of Hematology/Oncology, University of Illinois at Chicago, 840 South Wood Street, Chicago, IL, 60612, USA
[6] Genome Research Core, Research Resources Center, University of Illinois at Chicago

[*] Corresponding author, e-mail: evb@uic.edu

Source Data

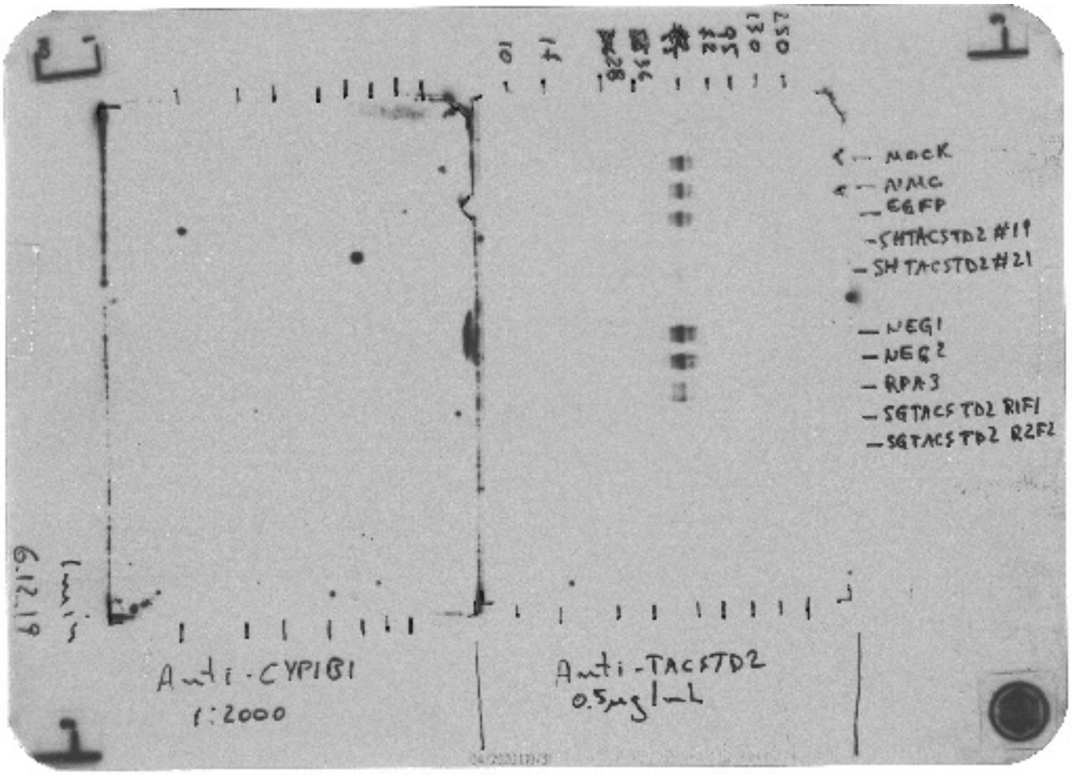

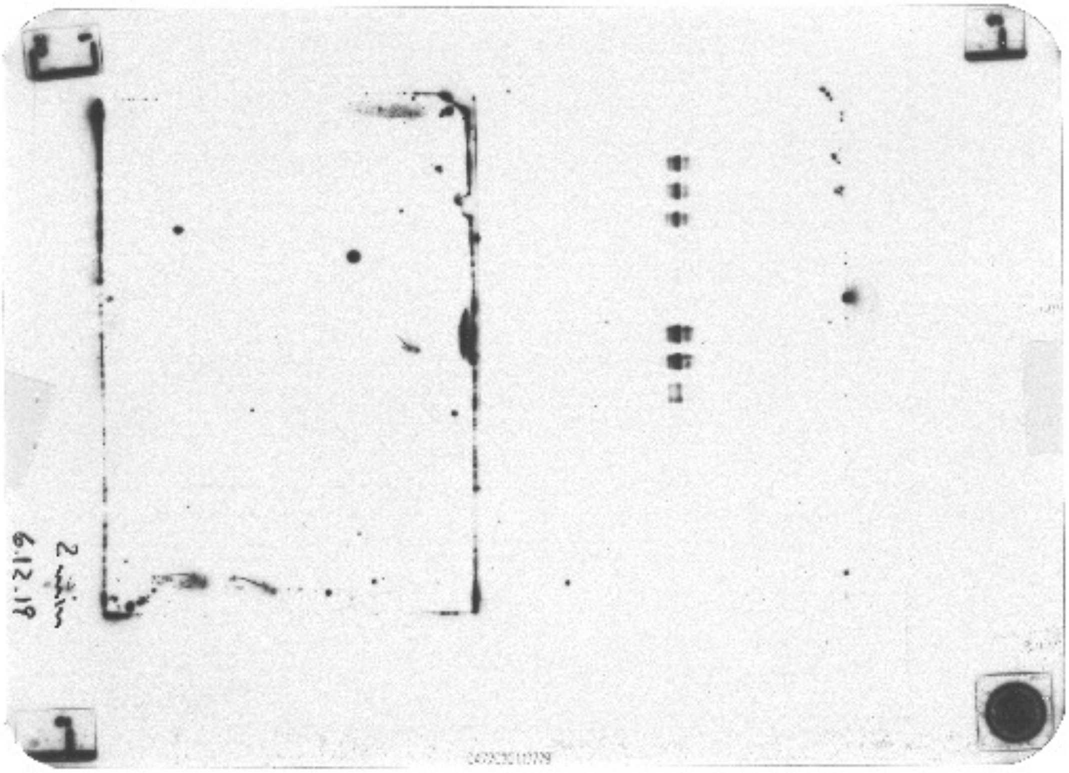

Source Data 1. Immunoblot analysis of cell lysates prepared from PC9 cells using antibodies to TACSTD2.

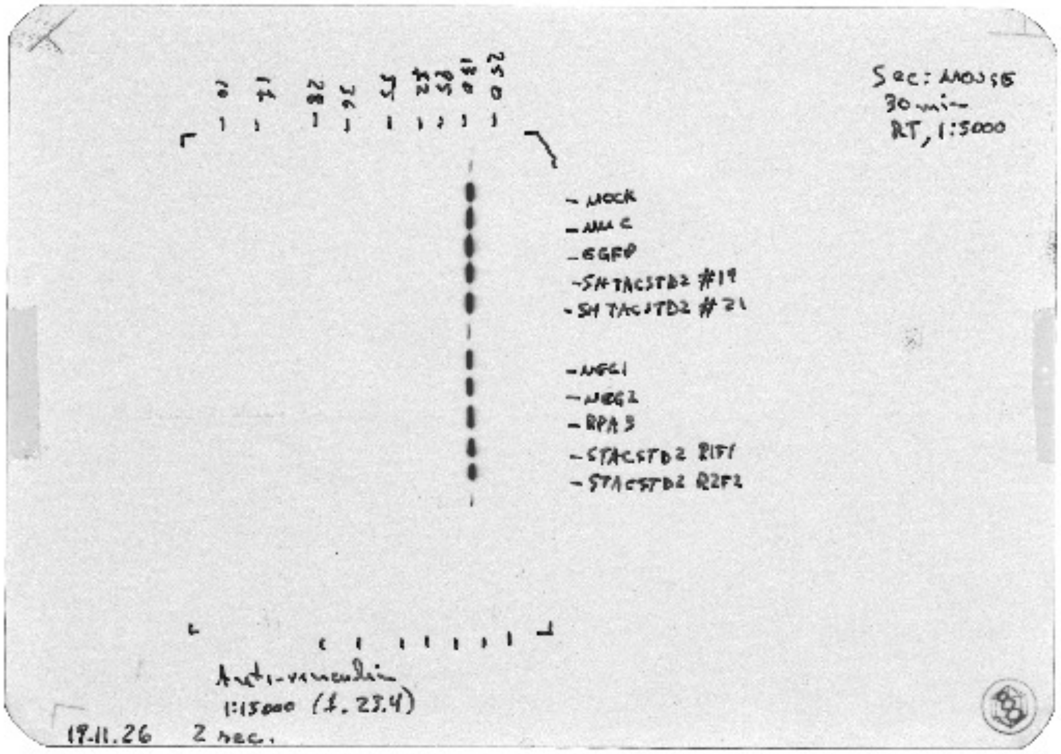

Source Data 2. Vinculin used as a loading control for the TACSTD2 experiment.

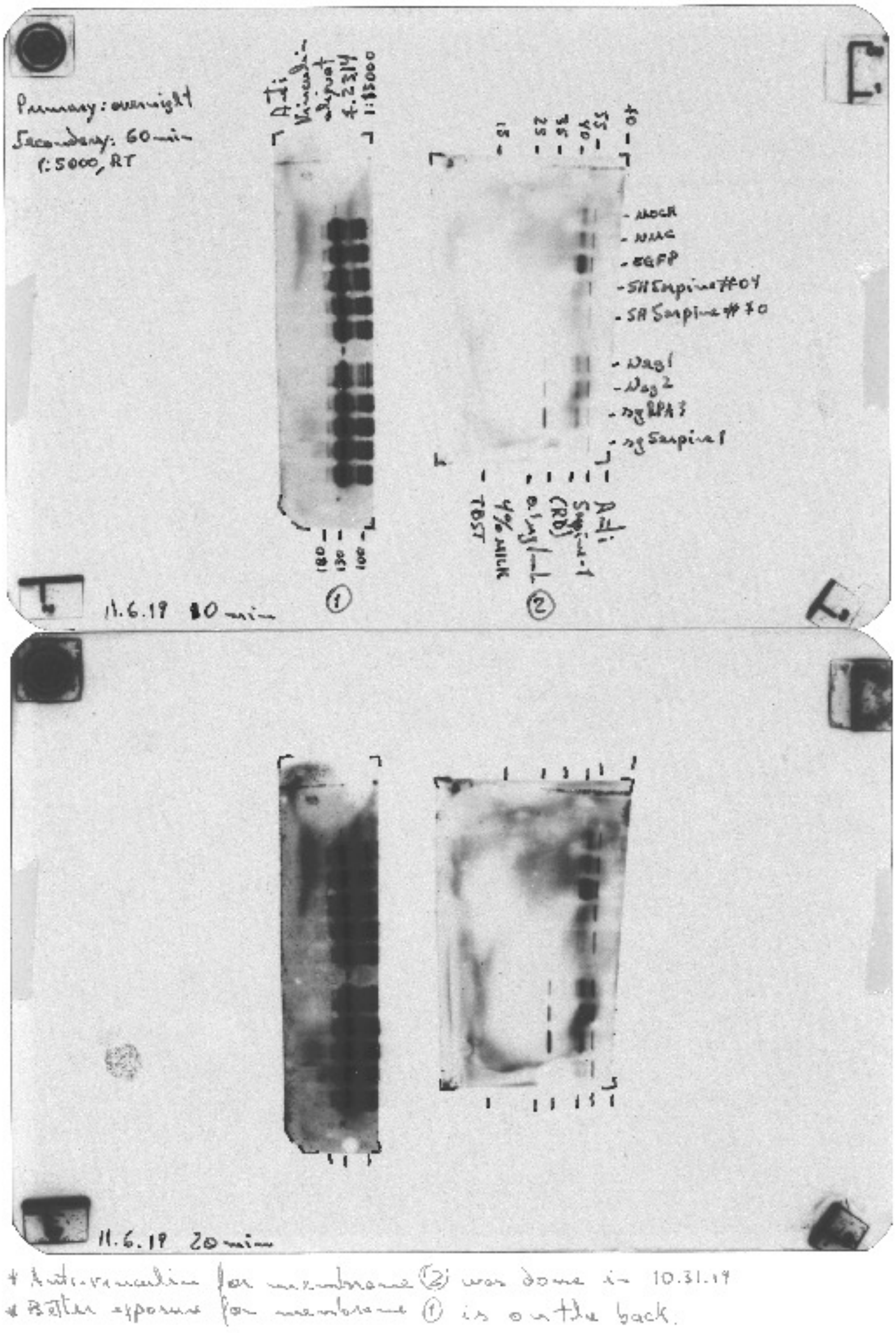

Source Data 3. Immunoblot analysis of cell lysates prepared from PC9 cells using antibodies to SERPINE1.

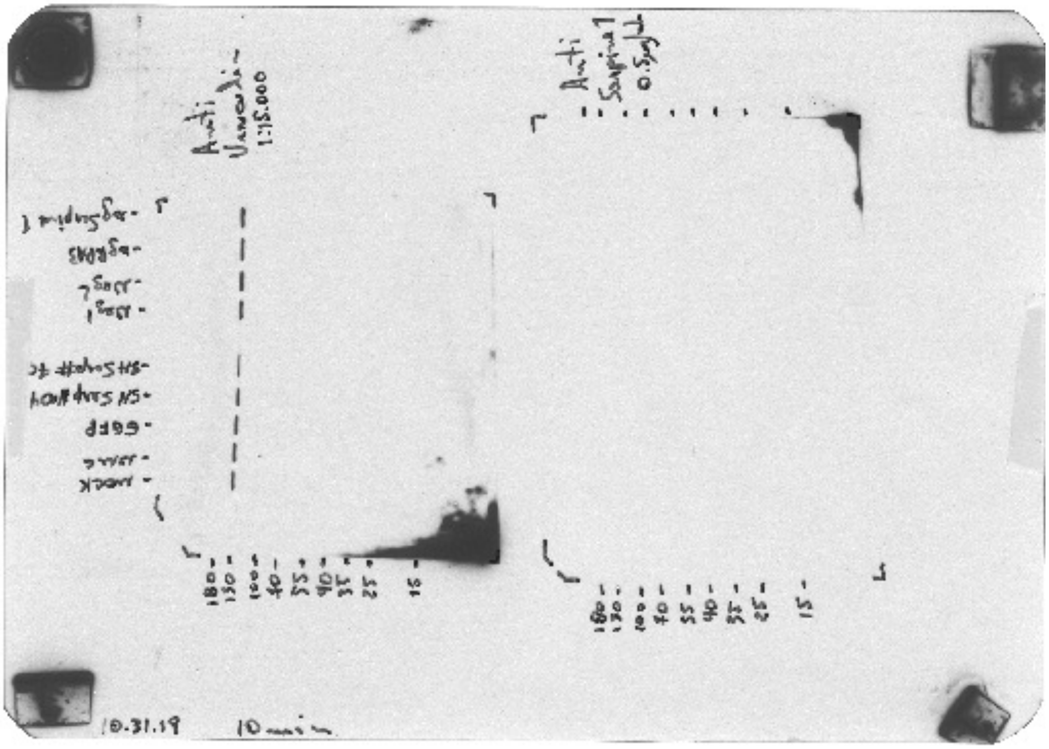

Source Data 4. Vinculin used as a loading control for the TACSTD2 experiment.
